# Balancing driving force, charge transport, and non-radiative recombination in organic solar cells with non-fused ring acceptors

**DOI:** 10.1039/d5el00136f

**Published:** 2025-11-10

**Authors:** Qian-Qian Zhang, Manasi Pranav, De-Li Ma, Bernhard Siegmund, Yuyao Xu, Yuming Wang, Melissa Van Landeghem, Hongzheng Chen, Chang-Zhi Li, Dieter Neher, Koen Vandewal

**Affiliations:** a State Key Laboratory of Silicon and Advanced Semiconductor Materials, Department of Polymer Science and Engineering, Zhejiang University Hangzhou 310027 P. R. China; b Hasselt University Agoralaan 1 3590 Diepenbeek Belgium koen.vandewal@uhasselt.be; c IMOMEC Division, IMEC Wetenschapspark 1 3590 Diepenbeek Belgium; d Energyville, IMO-IMOMEC Thorpark 8320 B-3600 Genk Belgium; e Institute of Physics and Astronomy, University of Potsdam Karl-Liebknecht Straße 24/25 14476 Potsdam Germany

## Abstract

The design of low-cost, stable, and high-efficiency non-fullerene acceptors requires a deeper understanding of the impact of the molecular structure on photovoltaic performance. In this study, we investigate the influence of gradual side-chain modifications of non-fused ring acceptors. The transition from non- (L0) to partially (L2) and fully chlorinated (L4) side chains enhances the molecular self-assembly, condenses the intermolecular packing, and balances the electron and hole mobility. Additionally, we observe lower bimolecular recombination coefficients and field-independent exciton dissociation upon gradual chlorination of the side chains, which improves the fill factor of the devices. However, the accompanying higher non-radiative voltage loss restricts the performance of the fully chlorinated L4 systems. Thus, the blend PM6:L2 balances efficient exciton dissociation with reduced non-radiative recombination, yielding the highest efficiency. This study emphasizes the pivotal role of side-chain halogenation in fine-tuning molecular packing and charge dynamics, offering guidelines for the next generation high efficiency photovoltaic materials.

## Abstract 2

Balancing driving force, charge transport, and non-radiative recombination in organic solar cells with non-fused ring acceptors through side-chain chlorination.

## Graphical abstract

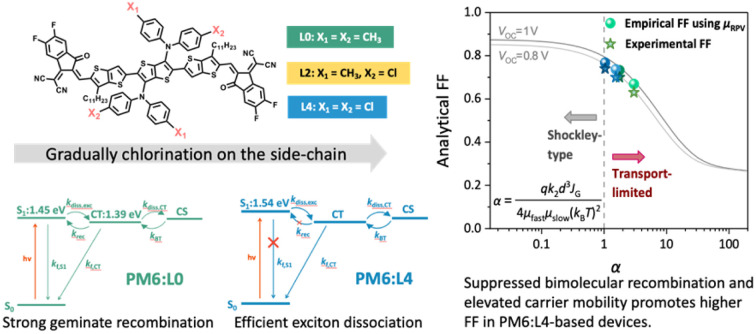

**Broader context**

As global efforts to achieve net-zero CO_2_ emissions intensify, organic solar cells (OSCs) are emerging as a promising technology with benefits like light weight, flexibility, and low CO_2_ footprint. While a wide range of high-performing OSC architectures has been reported in recent years, non-fused ring acceptors (NFRAs) are becoming increasingly attractive because of their competitive power conversion efficiencies and low synthetic complexity; however, the structure–photophysics relationships that govern their performance remain insufficiently understood. In this study, we combine structural, device-physics, and optical spectroscopic measurements and analytical modeling to identify the structural levers that control exciton dissociation and recombination in NFRA-based devices. We find that increasing side-chain chlorination of NFRAs improves intermolecular aggregation and suppresses the bimolecular recombination in blend films. At the same time, the increased exciton–charge-transfer (CT) energy enhances the driving force for charge generation, enabling efficient, field-independent exciton dissociation. However, higher degrees of chlorination also lead to increased non-radiative voltage losses. Among the investigated systems, the partially chlorinated L2 acceptor achieves the best balance between exciton dissociation efficiency and suppression of non-radiative recombination, resulting in the highest overall device efficiency. Our findings provide general guidelines for facilitating the development of efficient organic solar cells and support the broader advancement of sustainable solar energy technologies.

## 1 Introduction

As the world intensifies its efforts to achieve net-zero CO_2_ emissions, building-integrated organic solar cells (OSCs) are an emerging technology, promising light weight, mechanical flexibility, ultra-low CO_2_ footprint, and tunable visible transparency. A significant milestone in this field has been the introduction of non-fullerene acceptors (NFAs), which reduced the non-radiative voltage losses and boosted the power conversion efficiency (PCE) of OSCs from 11% (ref. 1) to over 21% within ten years.^2–7^ However, the construction of state-of-the-art fused ring acceptors, such as the Y6 series, requires chemically fusing the adjacent aromatics with covalent bonds that involve multiple-step synthesis and purification.^8,9^ The inevitable increase in synthetic complexity and energy input hinders a straight-forward translation of their high performance into mass production.^10–12^ To achieve efficient and stable NFAs with low synthetic complexity, we urgently need to deepen the understandings on the impact of the molecular structure on optoelectronic performance. In this respect, non-fused ring acceptors (NFRAs) are a promising absorber class: As their backbones contain no bridge atoms, multiple ring-fused reaction can be avoided,^13,14^ which lowers the synthetic complexity of NFRAs and allows for an effortless tailoring of their molecular structure.^15–19^ The PCEs of NFRAs based on the terthieno[3,2-*b*]thiophene backbone, named 2BTh–2F–C_2_, developed by Bo *et al.*, have reached up to 19%, comparable to fused-ring analogs.^20–22^

Progress in photovoltaic performance has been achieved by fine-tuning the molecular structure to adjust the material's absorption coefficient, spectral window, and energy levels to maximize the short-circuit current (*J*_SC_) and open-circuit voltage (*V*_OC_) of the device.^5,23–26^ Charge transport and recombination dynamics, determining the fill-factor (FF), are much harder to predict from the chemical structure.^27^ Low FFs indicate that the photocurrent depends strongly on the external bias, which corresponds to the internal electric field.^28^ Photocurrent generation in organic bulk-heterojunction solar cells involves at least three fundamental processes: (i) exciton dissociation to form an interfacial charge-transfer (CT) state, in competition with exciton decay; (ii) CT separation into free charges (FC), in competition with geminate CT recombination; and (iii) FC extraction, in competition with non-geminate recombination.^29^ Both the efficiency and dynamics of each step have been shown to depend on the electric field. Moreover, it was reported that the energetics and the morphology of the blend affect exciton dissociation, CT separation and charge collection in different ways. For example, the efficiency of exciton dissociation could be directly related to the HOMO offset,^30,31^ while for CT dissociation and charge collection, the morphology and especially the degree of phase separation and the molecular order within the individual domains seem to be of greater importance.^32^ Since the FF has an equal impact on PCE as *J*_SC_ and *V*_OC_, a more in-depth investigation into its relationship with chemical modifications through a detailed investigation of all photocurrent losses is essential for guiding the rational design of next-generation high-performance photovoltaic materials.

Previous work by some of the authors showed that modulating the halogen substituent on the aromatic side chains of terthieno[3,2-*b*]thiophene backbone-based NFRAs can tune intermolecular interactions and contribute to higher efficiency devices.^16^ However, the underlying relations between these structural characteristics, the photophysical mechanisms, and device performance are not clear. In this study, we synthesize a non-halogenated reference molecule, denoted as L0, and systematically compare it with its bi- and tetra-chlorinated analogs, L2 and L4 (corresponding to L1 in ref. 16), to elucidate the impact of side-chain chlorination on device physics. Two-dimensional grazing-incidence wide-angle X-ray scattering (2D-GIWAXS) measurements reveal enhanced molecular aggregation and improved π–π stacking with increasing chlorination, which reduces bimolecular recombination by an order of magnitude. On the other hand, time-delayed collection field (TDCF) measurements show that chlorination facilitates field-independent exciton dissociation and free charge generation through an increased HOMO offset in PM6:L*X* (*X* = 0, 2, 4) blend devices. Together, the optimized morphology and energy level alignment result in an improvement in the FF from PM6:L0 to PM6:L4. However, this comes at the cost of increased non-radiative voltage losses due to the larger energy offset in PM6:L4, which reduces the open-circuit voltage (*V*_OC_). Using an analytical diode model, we find that fast non-geminate recombination is the main factor limiting the PCE of PM6:L0, while slower carrier mobility also constrains the device performance. A compromise in the balance between exciton dissociation and non-radiative recombination losses is made with the partially chlorinated L2, which achieves the highest efficiency among the three acceptors, providing valuable insights for the design of next-generation, scalable NFAs.

## 2 Results and discussion

### 2.1. Optoelectronic properties and photovoltaic performances

**Fig. 1:**
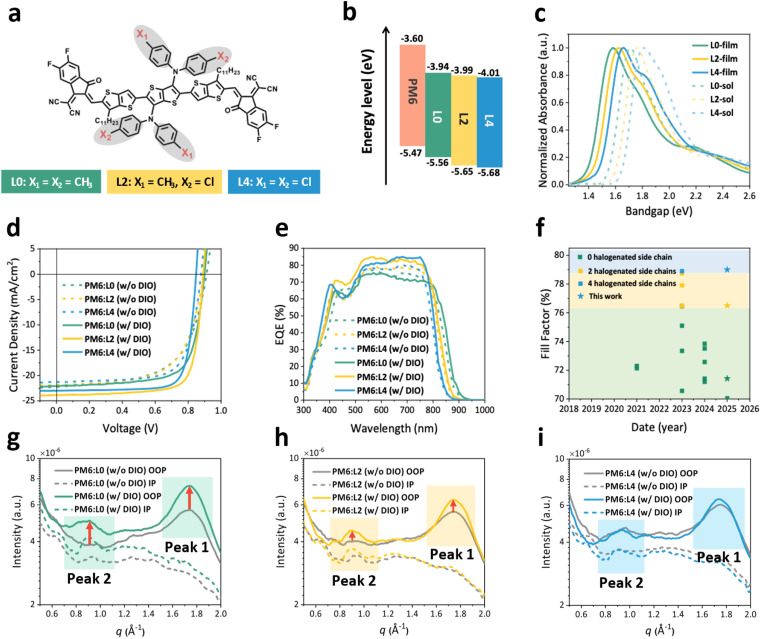
Molecular structure, opto-electronic, photovoltaic performance and morphology properties of L*X*. (a) The molecular structures of the L*X* acceptor molecules. (b) LUMO/HOMO levels were measured by cyclic voltammetry for L*X*. (c) The normalized absorbance of L*X* in chloroform and thin films. (d) Representative current–voltage (*J*–*V*) characteristics were measured under simulated AM1.5G for PM6 : L*X* (1 : 1.2) solar cells, processed with and without DIO in the casting solution. (e) EQE_PV_ curves for the PM6:L*X* devices. The slight discrepancy between *J*_SC,EQE_ and *J*_SC_ mainly arises from the different measurement conditions, and the small differences observed (≤5%) are within the typical experimental error range for OPV devices. (f) Fill-factors obtained in this work and in devices reported in the literature over the past four years, with a backbone as the case for L*X* (SI). An improvement of the FF upon halogenation is observed. (g–i) The GIWAXS intensity curves for PM6:L*X* blend films (solid line, OOP direction; dashed line, IP direction); higher crystallinity and new aggregated states emerge upon the addition of DIO to PM6:L0. No substantial change was observed for PM6:L4 when adding DIO.

Three homologous NFRAs with strong (L4),^16^ partial (L2),^16^ and absent chlorination (L0) of the side-chains were selected to study the impact of side-chain halogenation on photophysical mechanisms and their relation with photovoltaic performance (Fig. 1a, Scheme S1 in the SI and Fig. S1).^16,33^ The four phenylmethyl groups (P–Me) of the L0 side-chain *N*^3^′,*N*^3^′,*N*^6^′,*N*^6^′-tetra-*p*-tolyl 3′,6′-diamine, are gradually replaced by 2 (L2) or 4 (L4) chlorobenzene substituents (P–Cl). These subtle sidechain variations are found to notably affect the energetics and aggregation of the NFRAs.^16^ The trends in LUMO/HOMO levels from the cyclic voltammetry (CV) measurements show a downward shift of the energy levels (see Fig. S2 and Table S1), and agree well with earlier studies.^16^ The absorption spectra in solution and thin films are shown in Fig. 1c and Table S1. In solution, the absorption maxima for L0, L2, and L4 are located at 1.71 eV, 1.76 eV, and 1.82 eV, respectively. In thin films, the absorption maxima redshift, with L4 exhibiting the most blue-shifted absorption onset, which stems from the strong electron negativity of chlorine atoms, weakening the intramolecular charge transfer (ICT).^16^ However, the spectral shift from solution to thin films is the largest for L4, followed by L2 and L0, which we attribute to the enhanced intermolecular electrostatic interactions as reported recently for Y-type NFAs.^34,35^ Temperature-dependent absorption spectra (Fig. S3) further support this interpretation. Upon heating from 30 °C to 90 °C, all three acceptors show a band broadening and blue shift due to reduced pre-aggregation, but the effect is most pronounced for L0 (∼0.019 eV shift) and smaller for L2 and L4 (∼0.016 eV). This result indicates that side-chain chlorination enhances molecular pre-aggregation in solution. Consistently, density functional theory (DFT) calculations show that chlorination markedly increases the polarity of the side chains (Fig. S6), with the dipole moment increasing from 1.57 debye for the methyl-substituted template fragment to 4.28 debye for the chlorine-substituted fragment. The enhanced polarity provides a molecular origin for stronger intermolecular interactions and helps explain the largest solution-to-film redshift observed for L4.

The influence of the strong electron-deficient halogen atoms on photovoltaic performance was investigated by fabricating OSCs in a conventional configuration: indium tin oxide (ITO)/poly(3,4-ethylene dioxythiophene):poly(styrene-sulfonate) (PEDOT:PSS)/PM6 : NFRAs (1 : 1.2)/*N,N*′-bis(3-(3-(dimethylamino)propylamino)propyl)perylene-3,4,9,10-tetracarboxylic diimide (PDINN)/Ag. The optimized current density–voltage (*J*–*V*) curves, external quantum efficiency (EQE) curves and detailed photovoltaic parameters are displayed in Fig. 1d, e and Table 1, respectively. The *J*_SC_ and *V*_OC_ for the OSCs decrease upon chlorination, because of the blue shift in the absorption onset and descending LUMO energy levels (Fig. 1b). Intriguingly, the FF increases upon chlorination, from 0.60 for L0 to 0.64 for L2, reaching a peak of 0.72 for L4, leading to the highest PCE for L4.

**Table 1:** The photovoltaic parameters of OSCs under one sun equivalent illumination. (AM1.5G, 100 mA cm^−2^)

Parameters	PM6:L0 (w/o DIO)	PM6:L2 (w/o DIO)	PM6:L4 (w/o DIO)	PM6:L0 (w/DIO)	PM6:L2 (w/DIO)	PM6:L4 (w/DIO)
*V*_OC_ [V]	0.91 (0.90 ± 0.01)	0.90 (0.89 ± 0.01)	0.86 (0.85 ± 0.01)	0.91 (0.90 ± 0.01)	0.89 (0.88 ± 0.01)	0.84 (0.84 ± 0.01)
*J*_SC_ [mA cm^−2^]	22.2 (21.8 ± 0.2)	21.9 (21.9 ± 0.2)	21.3 (20.6 ± 0.8)	22.0 (22.2 ± 0.6)	24.0 (24.1 ± 0.2)	23.0 (22.9 ± 0.2)
*J*_SC,EQE_ [mA cm^−2^]^a^	21.1	20.8	20.0	21.2	22.8	21.8
FF	0.60 (0.58 ± 0.01)	0.64 (0.60 ± 0.02)	0.72 (0.71 ± 0.01)	0.71 (0.69 ± 0.01)	0.76 (0.76 ± 0.01)	0.79 (0.78 ± 0.01)
PCE [%]	12.1 (11.3 ± 0.4)	12.4 (11.6 ± 0.5)	13.3 (12.4 ± 0.6)	14.1 (13.8 ± 0.2)	16.5 (16.2 ± 0.2)	15.4 (15.0 ± 0.3)

a Calculated from the EQE curves.

To better understand the layer formation of the PM6:L*X* systems, we use a common high-boiling-point solvent additive 1,8-diiodooctane (DIO) to optimize the aggregate size of NFRAs.^36–40^ For PM6:L0, the *J*_SC_ and *V*_OC_ changed little after adding DIO. Meanwhile, for PM6:L2 and PM6:L4, the *V*_OC_ decreased slightly, and the *J*_SC_ increased by about 10%. The highest efficiency was achieved in the PM6:L2 system, reaching 16.5%, consistent with the literature.^16^ As the primary objective of this work is to explore the relationship between the material structure and photo physics mechanisms, we did not focus extensively on maximizing efficiency. Nevertheless, previous reports have demonstrated that the efficiencies of analogous NFRAs can be further improved to 17–19% through device optimization, underscoring their promising potential for future applications.^20–22,41^ Interestingly, the FF of the three devices increased significantly upon DIO treatment, while maintaining the trend of L0 < L2 < L4 (see Table 1). Comparing this DIO-treated FF trend with NFRA devices based on the same backbone as L*X*, we see a general trend of increased FFs upon increasing the number of halogenated side chains^15,16,20–22,33,42–46^ (see Fig. 1f and Table S2). Although selecting appropriate donor materials can enhance the FF of NFRA devices with no halogenated side chains, the FF did not exceed 0.77 and remained below those for NFRAs with halogenated side chains. This observation underlines a strong correlation between the FF of devices and the number of halogenated side chains in the NFRA structure which we will further explore in the following sections.

We employed two-dimensional grazing-incidence wide-angle X-ray scattering (2D-GIWAXS) to analyze the molecular stacking and orientation in PM6:L*X* blends and neat materials. In neat films, all three acceptors adopt a face-on orientation; however, L2 exhibits stronger crystallinity with a shorter π–π stacking distance (3.60 Å) compared to L0 (3.63 Å), whereas L4 shows excessive aggregation and poor solubility, resulting in weaker signals (Fig. S6). For the blend films, PM6:L2 (w/DIO) and PM6:L4 (w/DIO) were studied previously^16^ using GIWAXS, with similar results for those two systems. Here, we extend this study, by adding L0 and further focus on the influence of DIO. The resulting 2D scattering patterns and 1D intensity profiles, encompassing both in-plane (IP) and out-of-plane (OOP) directions, are illustrated in Fig. 1g–i and S5–S7, and the detailed parameters are listed in Table S3. In the IP direction, across all three systems, the overall intensity of signal peaks increases upon DIO addition, indicative of enhanced crystallization. In the OOP direction, PM6:L0 (w/o DIO) (grey line in Fig. 1g) exhibits a singular peak at 1.74 Å^−1^ (peak 1). Upon DIO treatment (green line in Fig. 1g), the intensity of peak 1 increases, accompanied by the emergence of a new peak at 0.91 Å^−1^, indicating the presence of a novel crystalized aggregated state. Similarly, PM6:L2 (w/o DIO) (grey line in Fig. 1h) shows peak 1 at 1.74 Å^−1^ and a secondary peak 2 with a minimal signal. Following DIO treatment, the intensities of both peak 1 and peak 2 increase, albeit to a lesser extent than L0. Conversely, the morphology of PM6:L4 is not strongly affected by the use of DIO (grey and blue lines in Fig. 1i do not strongly differ). In contrast, for L0 and L2, characterized by poorer self-aggregation properties, DIO supplementation facilitates the formation of long-range ordered domains. In summary, increasing the degree of chlorination of NFRAs stabilizes the ordered stacking of acceptors within the blends and reduces the system's reliance on additives like DIO.^16,39^

### 2.2. Non-geminate recombination and charge transport

**Fig. 2:**
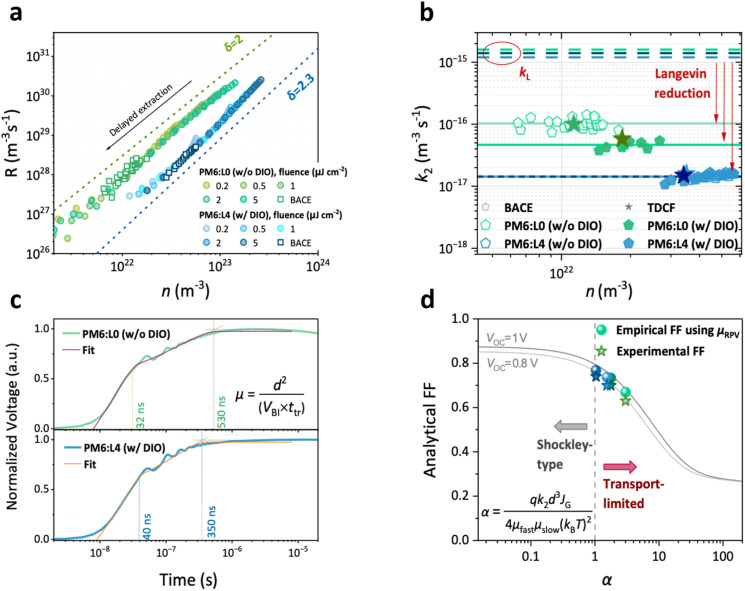
Non-geminate recombination and charge transport. (a) The recombination rate *R vs.* mobile charge carrier density *n* from time-delayed collection field (TDCF) and bias-assisted charge extraction (BACE) of PM6:L0 (w/o DIO) and PM6:L4 (w/DIO). Both systems show bimolecular recombination, with the slope *δ*, denoting the order of recombination, being close to 2. (b) The carrier-density-independent *k*_2_ values, as obtained from TDCF and BACE measurements, showing a reduction of bimolecular recombination with chlorination of the NFRA. The dotted lines indicate the *k*_2_ for Langevin-type encounter-limited recombination of free charge carriers, and the red arrows represent the Langevin reduction. (c) The RPV transients for PM6:L0 (w/o DIO) and PM6:L4 (w/DIO) are fitted as described in the SI, allowing the extraction of the transit times for fast and slow mobile charge carriers, as indicated in the inset. These transit times are related to the charge carrier mobility and active layer thickness (see the equation in the inset). (d) Analytical FF as a function of the figure-of-merit parameter *α*, compared with the experimentally obtained FF.

To investigate the influence of the improved morphology upon chlorination on the recombination pathways that influence the FF, we perform charge extraction techniques on full devices, namely TDCF and bias-assisted charge extraction (BACE).^29,47,48^ Herein, we probe the power-law dependence of the recombination rate of free charge carriers (*R*) on the mobile charge carrier density (*n*), to determine the recombination coefficient *k*. Fig. 2a (circular data points) shows *R-vs.-n* plots, as measured with TDCF for delayed extraction (*t*_del_) at various incident laser fluences for the L0- and L4-based systems. Based on the preceding discussion, the properties of L2 are expected to lie intermediately between those of L0 and L4. To emphasize the comparison, we present the *R-vs.-n* plots for the extreme cases (PM6:L0 (w/o DIO) and PM6:L4 (w/DIO)) in Fig. 2a, while the results for the other L0- and L4-blends are presented in Fig. S8–S10. Overlaid is the *R-vs.-n* plot as measured with BACE and shown with square data points. The dashed lines correspond to the linear fits to the data sets of both systems on a log–log scale, indicating the order of recombination. On choosing a fitting range that excludes leakage effects at low *n* and surface recombination effects at high *n*, both systems exhibit a dominant second-order dependence of *R* on *n*, and an ideality factor very close to *n*_id_ = 1 within a 10% error margin. This excludes trap-dominated loss pathways which would result in a lower recombination order. This indicates that non-geminate recombination in these systems occurs bimolecularly and is based on the Langevin-type encounter of mobile charge carriers. Using the power-law *R-vs.-n* relationship, the bimolecular coefficient *k*_2_ is calculated and plotted for the L0- and L4-based OSCs, processed w/o and with DIO, in Fig. 2b. The *k*_2_ values decrease progressively with increasing chlorination of side chains in the NFA: bimolecular recombination is almost a factor of 10 slower for L4-based OSCs than L0-based ones.

We attribute this reduction in *k*_2_ to the different and better aggregation of L4 as compared to L0 in blends with PM6. Previous reports show that improved aggregation of the constituent materials suppresses bimolecular recombination.^49,50^ Some reports relate this to the aggregation-assisted quadrupole effects that shift the energetics at the donor : acceptor interface, creating an energetic barrier for charge encounter.^51^ Besides the morphological influence, recently, *k*_2_ was observed to correlate with the ΔHOMO offset for a set of NFA-based OSCs, though the reason for the correlation is yet unknown.^30,52^ Also in our case, the L4 blend simultaneously features the largest ΔHOMO offset and lowest *k*_2_.

We now ascertain how reduced the recombination rates of these systems are relative to the Langevin recombination coefficient *k*_L_, *i.e.*, the upper limit for the bimolecular recombination coefficient *k*_2_ in low mobility semiconductors. According to Langevin's theory, the recombination of free charge carriers is related to their mean mobility, *i.e.* their speed of encounter. Deviations from this encounter-based loss mechanism are described by the Langevin reduction factor *γ* = *k*_2_/*k*_L_.^53^ To access the Langevin reduction factor we determine the charge carrier mobilities of the photogenerated charges, using the resistive photovoltage (RPV) technique at zero applied bias. Herein, we monitor the change in electrostatic potential when fast and slow photogenerated charge carriers transition across a working device. A transition between the slopes of the RPV voltage transient occurs for a significant difference in the transit times of the fast/slow charge carriers, which are shown by the dotted guidelines in Fig. 2c. The method to obtain the transit time is described in the SI. The mobility of the fast/slow charge carrier is calculated using the equation in Fig. 2c and the resulting values are listed in Table S4. To assign the fast and slow carriers observed in the RPV measurements to electrons and holes, steady-state space-charge-limited current (SCLC) measurements were performed on all blends, with the results summarized in Fig. S12 and Table S5. The data reveal that electrons are the faster carriers in all blends. The SCLC data also show that DIO treatment increases the mobility of the slower carrier, bringing it closer to that of the faster carriers, in agreement with the RPV analysis. However, the SCLC mobilities are overall lower than those from RPV, especially for the blends prepared w/o DIO, and are insufficient to explain the observed FFs. This discrepancy arises because SCLC probes injected carriers, which are more strongly affected by energetic disorder and interfacial barriers, whereas RPV reflects the transport of photogenerated charges under device-relevant conditions.^54^ Therefore, we have based further analysis on the mobilities from RPV. Interestingly, all blends have rather similar (imbalanced) mobilities, with slightly higher slow carrier mobilities when processing with DIO. This indicates that the improvement in the FF when going from PM6:L0 (w/o DIO) to PM6:L4 (w/DIO) comes from reduced recombination rather than faster extraction. Using the values of *k*_2_ and mobilities for the L0- and L4-based blends, we find that bimolecular recombination in these blends indeed deviates significantly from the Langevin encounter rate and that the deviation from the Langevin limit correlates with the DIO-induced aggregation noted from 2D-GIWAXS. The improved aggregation in the blends with DIO (or the lack thereof in the case of PM6:L4) relates to a smaller *k*_2_ and smaller *γ* as shown in Fig. 2b, which indeed aids the high FF of the L4-based OSCs.

To identify if we are approaching the maximum achievable FF, we use the figure-of-merit *α*, quantitatively relating transport and recombination parameters to the FF, as introduced by Neher *et al.*^55^ Herein, *α* depends on the photogenerated current (*J*_G_), the product of *µ*_fast_ and *µ*_slow_, *k*_2_, the active layer thickness (*d*), and the temperature (*T*) (see equation in Fig. 2d and SI Note). As *α* decreases to <1, one enters the Shockley-regime of transport, and the FF approaches its maximum value for a given *V*_OC_, in which transport losses do not limit the FF. Fig. 2d shows the FF-*vs.-α* plot for a *V*_OC_ of 0.8 V and 1 V. We overlaid the experimental FF, as well as calculated FF values of the L0- and L4-based systems using the RPV mobilities and *k*_2_ from the TDCF/BACE measurements described above. We observe a very good agreement between the experimental FF and empirical calculations. We further note that the PM6:L4 device with DIO is on the cusp of the transport-limited regime and the transport-limitless extraction regime (*α* = 1.04). For non-halogenated NFRAs the photocurrent becomes prone to the non-ideality of the OPV which is mainly due to the faster charge recombination, resulting in a lowering of the FF. This correlates with the reduced long-range order exhibited by L0-based systems and better molecular aggregation of L4-based systems as discussed previously. This further agrees with recent studies that showed enhanced ordered molecular packing in bulk heterojunction blends of PM6 with halogenated NFAs, with common reports of preserved molecular stacking, enhanced order (larger coherence lengths), and lower energy disorder.^56,57^ Although small in the present systems, we finally note that the FF is also affected by the field-dependence of free charge recombination, as shown in the following section.

### 2.3. Geminate recombination

**Fig. 3:**
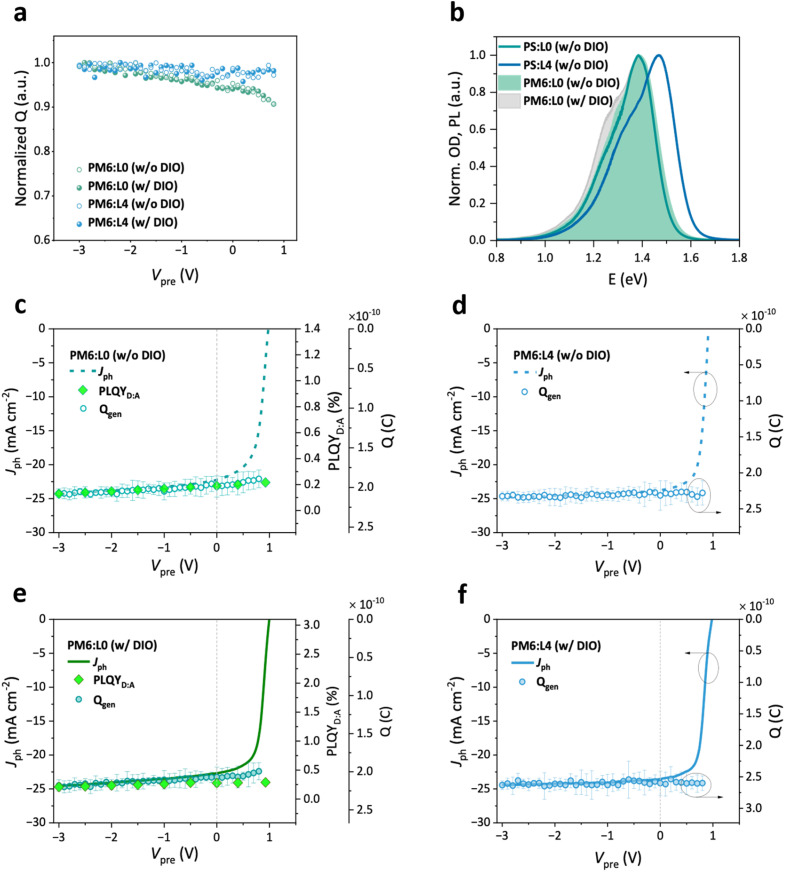
Geminate recombination analysis. (a) Normalized generated charges (*Q*_gen_) of L0 and L4 systems. L4 shows a completely field-independent generation. L0 has a marginal bias-dependence. (b) Normalized photoluminescence (PL) spectra of PS:L0 (w/o DIO) and PS:L4 (w/o DIO), overlapped with PL of PM6:L0 blends. The close resemblance of the L0-spectra indicates that the emission in L0-blends is governed by emission from the L0 singlet state. (c and e) Steady-state photocurrent from *J*–*V* measurement (*J*_ph_) overlapped with PLQY and *Q*_gen_ for the PM6:L0 systems, with and without DIO, respectively. The anti-correlation between the voltage dependence of photoluminescence quantum yield (PLQY) and of *Q*_gen_ indicates an incomplete singlet dissociation for L0, such that the competition between singlet decay and charge generation describes the field-dependence of the current–voltage characteristics (*J*–*V*) in L0 blends. (d and f) Overlap of *J*_ph_ with *Q*_gen_ for L4-systems, showing that the free charge carrier generation is already effective under short-circuit conditions.

**Fig. 4:**
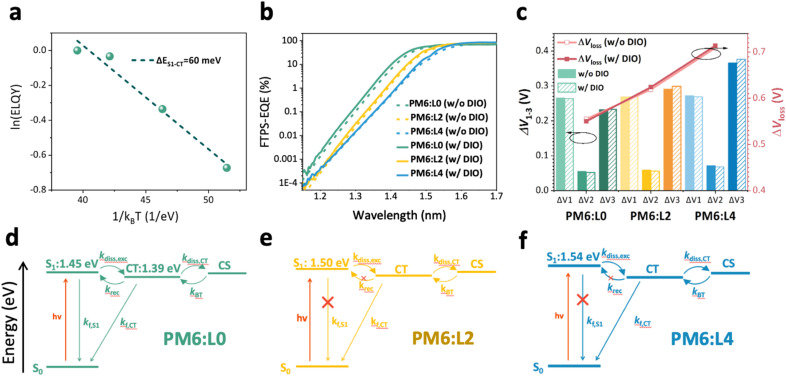
Voltage loss and charge generation-recombination processes. (a) Temperature-dependent electroluminescence quantum yield (ELQY-T) of PM6:L0. The slope gives the activation energy for charge-transfer (CT) states to emit a photon *via* the S_1_ state, *i.e.* Δ*E*_S_1_–CT_. (b) External quantum efficiency based on Fourier-transform photocurrent spectroscopy (FTPS-EQE) for PM6:L*X* devices. (c) A bar graph of the voltage losses for the PM6:L*X* blends. The red line represents the total voltage loss and the columns represent the detailed voltage losses. (d–f) The state diagrams of PM6:L*X* devices, indicating various transitions between the ground state singlet S_0_, singlet exciton S_1_, CT, and charge-separated (CS) states: photon absorption under illumination (*hν*), singlet decay (*k*_f,S_1__), exciton dissociation to CT (*k*_diss,exc_), reformation of the singlet exciton (*k*_rec_), CT state decay (*k*_f,CT_), CT state separation into free carriers (*k*_diss,CT_), and free carrier encounter to form CT (*k*_BT_).

We use TDCF measurements with <5 ns extraction delay to ascertain whether geminate losses during the free charge generation process limit the FF. Herein, the complete OSC device is excited with a *ca.* 5 ns laser pulse to photo generate a low density of charge carriers while under the influence of an applied bias (*V*_pre_). Then, a high reverse bias (here −4.5 V) rapidly extracts the photogenerated charge carriers, producing a transient photocurrent response (see Fig. S13 and S14). Under the condition that non-geminate recombination is absent during the delay and extraction, the integrated transient yields the generated charge *Q*_gen_. Fig. 3a shows the dependence of *Q*_gen_ on the pre-biasing conditions across the device during the generation process. Both PM6:L4 systems exhibit very efficient free charge generation, requiring no additional electric field to convert photoexcited singlet states into free charge. However, the L0-based blends exhibit a marginal bias-assistance of the free charge generation process, over a fairly wide bias range. We confirm with modified TDCF (see Fig. S14) that this bias-dependence arises only from geminate losses during free charge generation, indicating either an inefficient singlet exciton dissociation or charge transfer (CT) separation process (see ref. 58 for a detailed discussion of possible non-geminate recombination losses in classical and modified TDCF). The addition of DIO, although beneficial for the FF, does not affect this bias-dependence of free charge generation at all in either of the systems. As the addition of DIO significantly increases the crystallinity of the blend, this is somewhat surprising, as it suggests that the morphological change has no impact on the photon-to-free-charge conversion for L0. Although improved aggregation has been shown to enhance the CT separation process, we show in later parts of this section that CT separation appears to be efficient even without the DIO additive. The presence of a slight field-dependent free charge generation in the L0-based devices could arise from the shallower HOMO level of L0 as compared to L4, thereby reducing the driving force for CT formation through exciton dissociation.^58–60^ Importantly, the field-dependence of free charge generation in the L0- and L4-based OSCs fully describes the bias-dependence of the steady state photocurrent under short circuit conditions, as shown in Fig. 3c and 4f, which proves that non-geminate recombination currents play a negligible role in the shape of the photocurrent curve in this bias range. This no longer holds at positive biases, in part because increasing injection of charge carriers from the external circuits also determines the recombination currents that shape the bias-dependence of photocurrent approaching open-circuit.^47^

To pinpoint the sub-processes within free charge generation that are dominantly field-dependent in L0-based devices, we used a combination of bias-dependent steady-state photoluminescence (PL) of L0-based devices and PL quantum yield (PLQY) measurements.^60^ (see Fig. 3b, S15 and Table S6). We determined the PLQY experimentally using an integrating sphere, illuminating the sample at a 1 sun equivalent intensity under open-circuit conditions. In Fig. 3b, we show that the emission recorded from the PM6:L0 blends is attributed to L0 singlet emission in the blend, due to the close resemblance of the PL spectrum from the blend with the PL spectrum of polystyrene (PS):L0, *i.e.* L0 dispersed in an inert PS matrix (see Fig. 3b). The same is true for PM6:L4 blends (see Fig. S15d). We find that the PLQY of PM6:L4 blends does not change significantly with the addition of DIO. However, the PLQY of PM6:L0 marginally increases with DIO. The enhanced emission from PM6:L0 (w/DIO) arises from the improved PS:L0 emission with DIO, wherein the PLQY of the inert blend of L0 shows a *ca.* two-fold increase compared to that without DIO. On the other hand, PLQY values from PS:L4 blends with and without DIO do not differ significantly. These observations could be directly due to the improved aggregation of L0 in the presence of DIO, and the additive-indifferent aggregation properties of L4, as discussed previously. Compared to the emission of the NFRA in PS:L0 and PS:L4, the emission from the respective blends with PM6 is significantly quenched. Importantly, we further note a marginal reduction of the PM6:L0 blend emission under an applied bias with respect to the acceptor singlet decay.

The PL spectra of the L0-blends are then referenced to the PL quantum yield of the PM6:L0 blends to obtain a bias-dependent PLQY, *i.e.* PLQY(V). The procedure for this conversion is described in detail in ref. 53. We plot the PLQY(V) data of PM6:L0 (w/o DIO) over the same voltage range as the TDCF data, assuming that complete generation results in zero-emission, and that zero generation would give rise to a blend emission equaling the PLQY of the NFA. In Fig. 3c and e, we compare the bias-dependent PLQY and TDCF generation data with the steady state photocurrent from *J*–*V* measurements for the PM6:L0 blends, which perfectly overlap. Importantly, the inverse quantitative correlation between the *Q*_gen_ from TDCF and PLQY_D:A_ from PL over the full bias range means that an increase in the free charge generation comes at the cost of a reduction of PLQY(V) of PM6:L0. In other words, regardless of the processing conditions of the blend, exciton recombination is the main if not only competing process for free charge generation^60^ and not CT separation. This conclusion is supported by the fact that the PLQY of the blend (extrapolated to zero-generation) is equal to the PLQY of the acceptor in an inert PS matrix (PLQY_PS:L0_ in Fig. 3b), meaning that the PL-signal in the bias-dependent experiments is primarily from radiative singlet decay, and not due to the radiative recombination of the CT state through, for instance, intensity-borrowing. Following the same line of argument, we do not see evidence for singlet–CT hybridization, which would affect the singlet and CT emission strengths.^61,62^ We finally note that while field-dependent free charge generation would in general contribute to the FF loss,^60^ given the marginal bias-dependence in the L0-based devices, the contribution of these geminate losses to FF losses is expectably minor compared to changes in recombination due to modified aggregation.

### 2.4. Energy loss and optoelectronic process analysis

Previous reports have shown that inefficient singlet exciton dissociation into CT states becomes indeed problematic in OSC blends with a diminishing offset between the singlet state (S_1_) and CT energy states.^60,63–66^ Therefore, it becomes important to ascertain the magnitude of this S_1_–CT driving force, Δ*E*_S_1_–CT_ = *E*_S_1__ − *E*_CT_. A common approach to determine this driving force is to use the HOMO offset, which however, oversimplifies the situation.^66,67^ We, therefore, determine the S_1_–CT offset in L0-based blends using the temperature-dependent electroluminescence approach.^52^ Herein, we consider that repopulation of S_1_ states from free charges occurs *via* the reformation of the interfacial CT states, which either decays to the ground state or back-transfer to the S_1_ through a thermally stimulated process. In Fig. 4a, we plot the natural logarithm of the electroluminescence quantum yield (ELQY), normalized to room temperature against the inverse of *k*_B_*T*, whereas the slope provides the thermal activation for S_1_ reformation from CT, *i.e.* the S_1_–CT energy offset (see Fig. S16). A Δ*E*_S_1_–CT_ of 60 meV is recorded and differs from the ΔHOMO offset of 90 meV, obtained when subtracting the CV-based HOMO values. This is likely due to the difference in S_1_ and CT binding energy, among other effects.^60^ In light of this offset of 60 meV being a few *k*_B_*T*, it is interesting that L0-based devices still achieve such efficient free charge generation, with only marginal field dependence, in contrast to other previously reported blends with similar S_1_–CT offsets but significantly hindered photon-to-charge conversion.^60^ Given the very low ELQY of PM6:L4 blends, we could not obtain reportable temperature-dependent ELQY data for L4-blend systems. This is likely due to the significantly higher S_1_–CT offset in L4-blends, which is caused by the higher energy of the S_1_ in connection with the deeper LUMO of L4 and related to this smaller CT energy. On the other hand, this higher offset is beneficial for efficient free charge generation as seen in the L4-based blends.1
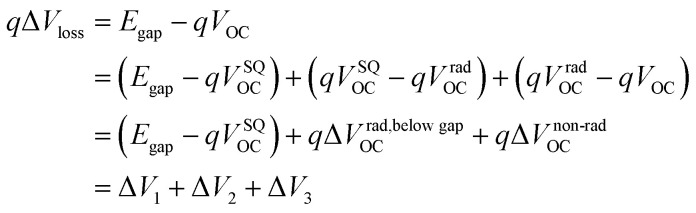


The improvement in FF upon chlorination comes at the cost of a significant reduction in *V*_OC_ from L0 to L4, a trade-off also observed in previous studies.^31^ To assess the voltage loss in the three systems, we employed high-sensitivity Fourier-transform photocurrent spectroscopy (FTPS) and ELQY and utilized the Shockley–Queisser (S–Q) limit theory to analyze them;^68–71^ the calculation procedure is presented in the SI. The total voltage loss, which is quantified as *q*Δ*V*_OC_, can be attributed to three main factors, see eqn (1):

As shown in Fig. 4b, c, Table 2 and Fig. S17, we find the radiative recombination above the gap, *q*Δ*V*_1_, to vary little, with values 264 and 272 mV across all six samples. Meanwhile, the radiative recombination below the gap, *q*Δ*V*_2_, progressively increases from L0, *via* L2 to L4. While experiencing the most severe geminate recombination due to the singlet decay, the PM6:L0 systems also show the highest ELQY in the order of magnitude of 0.1% (Fig. S17b). Consequently, this system exhibits the lowest non-radiative recombination voltage loss of Δ*V*_3_ = 233 mV, summing up to a total voltage loss of 555 mV. While the PM6:L4 systems display a high exciton dissociation yield, the elevated Δ*V*_3_ of 366 mV limits their photovoltaic performance. Previous studies suggest that increasing the CT state energy or reducing the energy gap between CT and singlet states can decrease the non-radiative voltage loss Δ*V*_3_.^72^ This finding is supported by our EL spectra (Fig. S17a), which show that in blend films with a low energy offset (L0 systems), CT emissions are overshadowed by dominant singlet emissions. The calculated *V*_OC_ (*V*_OC,cal_) aligns well with the measured *V*_OC_ (*V*_OC,mea_) from experiments, validating this method for quantifying *V*_OC_ losses. The losses Δ*V*_2_ and Δ*V*_3_ substantially increase upon acceptor chlorination, translating into an increase in the total voltage loss of 64 mV (L2) and 154 mV (L4), compared to L0 (see Δ*V*_1_, Δ*V*_2_, Δ*V*_3_, and total Δ*V*_loss_ in Fig. 4c). Although the chlorinated side chains facilitate the ordered packing of L4, the reduced frontier orbital energy levels increase the energy offset between PM6 and L4, thereby increasing the voltage loss.

**Fig. 5:**
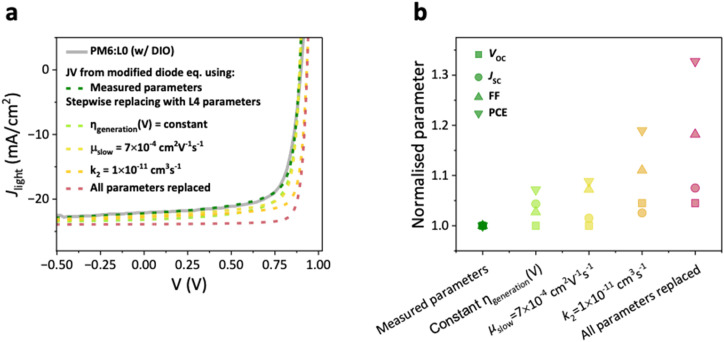
(a) Experimental *JV* curve of PM6:L0 (w/DIO), described by using the diode equation from Sandberg *et al.* extended with a field bias-dependent free charge generation term (green dotted line). The input parameters to the diode equation are the experimentally determined bias-dependence of photocurrent, bimolecular recombination coefficient *k*_2_ and charge mobility *µ*. Also plotted are the predicted *JV*-characteristics shown with the light green to red dotted lines, simulated with stepwise increments in the input parameters closer to those of PM6:L4. (b) The normalized corresponding photovoltaic metrics of all *J*–*V* curves produced with the Sandberg diode model, using the measured input parameters and hypothetical input parameters as indicated in the *x*-axis.

**Table 2:** Voltage loss profiles of OSCs based on PM6:L*X*

	PM6:L0 (w/o DIO)	PM6:L2 (w/o DIO)	PM6:L4 (w/o DIO)	PM6:L0 (w/DIO)	PM6:L2 (w/DIO)	PM6:L4 (w/DIO)
*E* ^PV^_g_ (V)	1.46	1.51	1.54	1.45	1.50	1.54
Δ*V*_1_ (mV)	266	269	272	264	269	269
Δ*V*_2_ (mV)	56	59	71	52	56	68
Δ*V*_3_ (mV)	233	291	366	233	299	377
Δ*V* (mV)	555	619	709	550	624	714
*V*_OC,cal_ (V)	0.91	0.89	0.83	0.90	0.88	0.82
EQE_EL_ (%)	1.1 × 10^−4^	1.3 × 10^−5^	7.2 × 10^−7^	1.2 × 10^−4^	9.4 × 10^−6^	4.7 × 10^−7^

We summarize the optoelectronic dynamics of the PM6:L*X* blends, as illustrated in the state diagrams in Fig. 4d–f. For PM6:L0, we find a minimal energy difference between the S_1_ state and the CT state of 60 meV. This low driving force configuration facilitates a high rate of recombination from the CT state to the S_1_ state (*k*_rec_), establishing a dynamic equilibrium with the exciton dissociation (*k*_diss,exc_) process and brings high PL recombination intensity for the L0 systems. Upon applying a bias, more charge carriers were extracted to the external circuit, and the equilibrium shifts towards the charge separation and exciton dissociation, reducing S_1_ state recombination and lowering the PL intensity. Conversely, for chlorinated PM6:L4, the large offset in HOMO energy between PM6 and L4 allows excitons to dissociate effectively, even in the absence of an electric field. Consequently, applying an external electric field yields no gain in exciton dissociation, and the PL intensity remains relatively low and stable. Although the ΔHOMO in PM6:L2 systems is not as high as that in PM6:L4 systems, the 3D stacking mode of acceptors reduces the exciton binding energy, providing sufficient driving force for exciton dissociation (Fig. S18) and relatively low voltage loss within the system.^73^ We find the highest photovoltaic performance for an intermediate HOMO level offset, as in our case for DIO-treated PM6:L2. Hence, moderate halogenation of the acceptor side chains allows balancing the driving force for charge-transfer formation and non-radiative voltage losses within the system.

Given the fact that the non-chlorinated PM6:L0 blend has the lowest voltage loss, we finally ask the question how much the PCE of this system suffers from field-dependent free charge generation and extraction losses. To this end, we reproduced the experimental *JV*-curves of the L0 and L4 blends with a recently proposed diode model equation.^74^ This model considers bimolecular recombination between photogenerated charge carriers as well as between photogenerated and dark-injected charge as loss channels. It has been further modified to consider bias-dependence of free charge generation. The measured values of charge mobility (from RPV), bias-dependence of free charge generation (from TDCF), and recombination coefficients (from BACE and TDCF) were used as input parameters in the model. The resulting analytical *J*–*V* curves agree very well with the experimental *J*–*V* curves. This is shown for the DIO-processed PM6:L0 blend in Fig. 5a by the grey solid line and green dotted line (see Fig. S19 for all four blends). This proves to us that the analytical model captures all geminate and non-geminate losses in our devices. We then checked how a stepwise increment in the input parameters closer to those of PM6:L4 would help the PCE, as shown in the dotted lines in Fig. 5a and the corresponding normalized *J*–*V* parameters in Fig. 5b. We find that field-dependent generation plays a minor role affecting the FF in these NFRA-based devices, although this has the most impact on the device *J*_SC_. On the other hand, reducing bimolecular recombination causes a great improvement of the FF, but also increases *V*_OC_. We finally tested the importance of the slower mobility on the device performance. Both experimental and simulation studies have consistently shown that the extraction–recombination balance determining the FF is largely governed by the value of the slower carrier mobility, while it is only weakly affected by the faster carrier mobility.^75^ In line with this, we observed a significant improvement of the FF when increasing the slower carrier mobility to 7 × 10^4^ cm^2^ V^−1^ s^−1^, whereas the other photovoltaic parameters remained nearly unchanged. Combining these improvements could yield a predicted PCE of 18.9%, stemming primarily from the improved FF *via* a better transport-recombination balance.

## 3 Conclusion

This study investigates the impact of systematical chlorination of NFRA side chains—from L0 to L4—on the photophysical behavior and device performance of organic solar cells with non-fused ring acceptors. The enlarged energy offset between the local exciton and the interfacial CT state upon chlorination enables highly efficient, field-independent exciton dissociation, indicating the critical role of interfacial energetics in driving free charge generation. Concurrently, enhanced molecular aggregation improves blend morphology, reduces the bimolecular recombination coefficient by nearly an order of magnitude, and facilitates efficient charge collection. As a result, PM6:L4 devices processed with DIO achieve FFs approaching 80%, with the FF trend (L0 < L2 < L4) consistent with previous observations for halogenated NFRAs. However, downshifting the frontier orbital energy levels of the NFRAs upon chlorination also lowers the energy gap and suppresses the reformation of the emissive NFA exciton, thus increasing non-radiative energy losses. Consequently, L2 with an intermediate offset in the HOMO level balances the driving force for CT state formation and non-radiative recombination loss, allowing for the highest efficiency. We, finally tested how the PM6:L0 (w/DIO) blend with the smallest voltage loss would benefit from reduced geminate and non-geminate losses. Here, fast non-geminate recombination was found to dominate the PCE loss. Our *J*–*V* simulations also showed that a further increase in the slower carrier mobility simulated a FF above 80%, and a PCE toward 19% for these materials with low synthetic complexity. This study provides valuable insights into the structure–performance relationship of non-fused ring electron acceptors and is expected to contribute to the further development of scalable high-efficiency photovoltaic materials.

## Author contributions

Q.-Q. Z. and K. V. conceived the ideas. Q.-Q. Z. and D.-L. M. designed the L*X* acceptors under the supervision of C.-Z. L. and H. C.; D.-L. M. synthesized the acceptors, and conducted the UV-vis, CV, MALDL-TOF MS, and NMR characterization studies. Q.-Q. Z. made the devices and carried out the *J*–*V*, EQE_PV_, EQE_EL_, and FTPS-EQE characterization studies under the supervision of K. V.; M. P. carried out the measurements of BACE, TDCF, RPV, temperature-dependent EL, steady state PL (zero-field and biased PL), and PLQY and performed the diode model analysis under the supervision of D. N.; Y. X. prepared the samples for 2D-GIWAXS under the supervision of C.-Z. L.; Q.-Q. Z. and Y. W. conducted the bias dependent PL measurements. Q.-Q. Z. and M. V. L. carried out the EL measurements. Q.-Q. Z., M. P., B. S., C.-Z. L., D. N., and K. V. analyzed the experimental results. Q.-Q. Z., M. P., B. S., C.-Z. L., D. N., and K. V. wrote the manuscript. K. V., D. N. and C.-Z. L. supervised the project. All authors discussed the results and commented on the final manuscript.

## Conflicts of interest

There are no conflicts to declare.

## Supplementary Material

EL-002-D5EL00136F-s001

## Data Availability

The data that support the findings of this study are available within the article and its supplementary information (SI). Supplementary information: materials and methods, synthesis details, supplementary figures, supplementary tables, *etc.* See DOI: https://doi.org/10.1039/d5el00136f.

## References

[cit1] Zhao J., Li Y., Yang G., Jiang K., Lin H., Ade H., Ma W., Yan H. (2016). Nat. Energy.

[cit2] Sun Y., Wang L., Guo C., Xiao J., Liu C., Chen C., Xia W., Gan Z., Cheng J., Zhou J., Chen Z., Zhou J., Liu D., Wang T., Li W. (2024). J. Am. Chem. Soc..

[cit3] Yuan J., Zhang Y., Zhou L., Zhang G., Yip H.-L., Lau T.-K., Lu X., Zhu C., Peng H., Johnson P. A., Leclerc M., Cao Y., Ulanski J., Li Y., Zou Y. (2019). Joule.

[cit4] Zhu L., Zhang M., Zhou Z., Zhong W., Hao T., Xu S., Zeng R., Zhuang J., Xue X., Jing H., Zhang Y., Liu F. (2024). Nat. Rev. Electr. Eng..

[cit5] Lin Y., Wang J., Zhang Z. G., Bai H., Li Y., Zhu D., Zhan X. (2015). Adv. Mater..

[cit6] Zhao W., Li S., Yao H., Zhang S., Zhang Y., Yang B., Hou J. (2017). J. Am. Chem. Soc..

[cit7] Li C., Cai Y., Hu P., Liu T., Zhu L., Zeng R., Han F., Zhang M., Zhang M., Lv J., Ma Y., Han D., Zhang M., Lin Q., Xu J., Yu N., Qiao J., Wang J., Zhang X., Xia J., Tang Z., Ye L., Li X., Xu Z., Hao X., Peng Q., Liu F., Guo L., Huang H. (2025). Nat. Mater..

[cit8] Yu Z. P., Liu Z. X., Chen F. X., Qin R., Lau T. K., Yin J. L., Kong X., Lu X., Shi M., Li C. Z., Chen H. (2019). Nat. Commun..

[cit9] Shi Y., Chang Y., Lu K., Chen Z., Zhang J., Yan Y., Qiu D., Liu Y., Adil M. A., Ma W., Hao X., Zhu L., Wei Z. (2022). Nat. Commun..

[cit10] Machui F., Hösel M., Li N., Spyropoulos G. D., Ameri T., Søndergaard R. R., Jørgensen M., Scheel A., Gaiser D., Kreul K., Lenssen D., Legros M., Lemaitre N., Vilkman M., Välimäki M., Nordman S., Brabec C. J., Krebs F. C. (2014). Energy Environ. Sci..

[cit11] Ma Y., Cai D., Wan S., Wang P., Wang J., Zheng Q. (2020). Angew. Chem., Int. Ed..

[cit12] Wang G., Wang J., Cui Y., Chen Z., Wang W., Yu Y., Zhang T., Ma L., Xiao Y., Qiao J., Xu Y., Hao X. T., Hou J. (2024). Angew. Chem., Int. Ed..

[cit13] Ma L., Zhang S., Zhu J., Wang J., Ren J., Zhang J., Hou J. (2021). Nat. Commun..

[cit14] Wang Y., Yang M., Chen Z., Zhong J., Zhao F., Wei W., Yuan X., Zhang W., Ma Z., He Z., Liu Z., Huang F., Cao Y., Duan C. (2025). Nat. Commun..

[cit15] Li D., Zhang H., Cui X., Chen Y. N., Wei N., Ran G., Lu H., Chen S., Zhang W., Li C., Liu Y., Liu Y., Bo Z. (2024). Adv. Mater..

[cit16] Ma D. L., Zhang Q. Q., Li C. Z. (2023). Angew. Chem., Int. Ed..

[cit17] Yang N., Cui Y., Xiao Y., Chen Z., Zhang T., Yu Y., Ren J., Wang W., Ma L., Hou J. (2024). Angew. Chem., Int. Ed..

[cit18] Yang N., Cui Y., Zhang T., An C., Chen Z., Xiao Y., Yu Y., Wang Y., Hao X. T., Hou J. (2024). J. Am. Chem. Soc..

[cit19] Wen T. J., Liu Z. X., Chen Z., Zhou J., Shen Z., Xiao Y., Lu X., Xie Z., Zhu H., Li C. Z., Chen H. (2021). Angew. Chem., Int. Ed..

[cit20] Gu X., Zeng R., He T., Zhou G., Li C., Yu N., Han F., Hou Y., Lv J., Zhang M., Zhang J., Wei Z., Tang Z., Zhu H., Cai Y., Long G., Liu F., Zhang X., Huang H. (2024). Adv. Mater..

[cit21] Han Z., Zhang C., He T., Gao J., Hou Y., Gu X., Lv J., Yu N., Qiao J., Wang S., Li C., Zhang J., Wei Z., Peng Q., Tang Z., Hao X., Long G., Cai Y., Zhang X., Huang H. (2024). Angew. Chem., Int. Ed..

[cit22] Zeng R., Zhang M., Wang X., Zhu L., Hao B., Zhong W., Zhou G., Deng J., Tan S., Zhuang J., Han F., Zhang A., Zhou Z., Xue X., Xu S., Xu J., Liu Y., Lu H., Wu X., Wang C., Fink Z., Russell T. P., Jing H., Zhang Y., Bo Z., Liu F. (2024). Nat. Energy.

[cit23] Baran D., Gasparini N., Wadsworth A., Tan C. H., Wehbe N., Song X., Hamid Z., Zhang W., Neophytou M., Kirchartz T., Brabec C. J., Durrant J. R., McCulloch I. (2018). Nat. Commun..

[cit24] Li C., Zhou J., Song J., Xu J., Zhang H., Zhang X., Guo J., Zhu L., Wei D., Han G., Min J., Zhang Y., Xie Z., Yi Y., Yan H., Gao F., Liu F., Sun Y. (2021). Nat. Energy.

[cit25] Janssen R. A., Nelson J. (2013). Adv. Mater..

[cit26] Jiang K., Zhang J., Zhong C., Lin F. R., Qi F., Li Q., Peng Z., Kaminsky W., Jang S.-H., Yu J., Deng X., Hu H., Shen D., Gao F., Ade H., Xiao M., Zhang C., Jen A. K. Y. (2022). Nat. Energy.

[cit27] Xiao B., Calado P., MacKenzie R. C. I., Kirchartz T., Yan J., Nelson J. (2020). Phys. Rev. Appl..

[cit28] Gupta D., Mukhopadhyay S., Narayan K. S. (2010). Sol. Energy Mater. Sol. Cells.

[cit29] Kurpiers J., Ferron T., Roland S., Jakoby M., Thiede T., Jaiser F., Albrecht S., Janietz S., Collins B. A., Howard I. A., Neher D. (2018). Nat. Commun..

[cit30] Tokmoldin N., Sun B., Moruzzi F., Patterson A., Alqahtani O., Wang R., Collins B. A., McCulloch I., Lüer L., Brabec C. J., Neher D., Shoaee S. (2023). ACS Energy Lett..

[cit31] Zhang X., Yao N., Wang R., Li Y., Zhang D., Wu G., Zhou J., Li X., Zhang H., Zhang J., Wei Z., Zhang C., Zhou H., Zhang F., Zhang Y. (2020). Nano Energy.

[cit32] Zhang X., Li C., Xu J., Wang R., Song J., Zhang H., Li Y., Jing Y.-N., Li S., Wu G., Zhou J., Li X., Zhang Y., Li X., Zhang J., Zhang C., Zhou H., Sun Y., Zhang Y. (2022). Joule.

[cit33] Wang X., Lu H., Liu Y., Zhang A., Yu N., Wang H., Li S., Zhou Y., Xu X., Tang Z., Bo Z. (2021). Adv. Energy Mater..

[cit34] Liu W., Andrienko D. (2023). J. Chem. Phys..

[cit35] Giannini S., Sowood D. J. C., Cerdá J., Frederix S., Grüne J., Londi G., Marsh T., Ghosh P., Duchemin I., Greenham N. C., Vandewal K., D’Avino G., Gillett A. J., Beljonne D. (2024). Mater. Today.

[cit36] McDowell C., Abdelsamie M., Toney M. F., Bazan G. C. (2018). Adv. Mater..

[cit37] Zhang X., Wang H., Li D., Chen M., Mao Y., Du B., Zhuang Y., Tan W., Huang W., Zhao Y., Liu D., Wang T. (2020). Macromolecules.

[cit38] Zhao Q., Lai H., Chen H., Li H., He F. (2021). J. Mater. Chem. A.

[cit39] Chen L., Ma R., Yi J., Dela Peña T. A., Li H., Wei Q., Yan C., Wu J., Li M., Cheng P., Yan H., Zhang G., Li G. (2023). Aggregate.

[cit40] Li D., Zhang X., Liu D., Wang T. (2020). J. Mater. Chem. A.

[cit41] Ye S., Chen T., Yu J., Wang S., Li S., Wang J., Fu Y., Zhu Y., Wang M., Lu X., Ma Z., Li C.-Z., Shi M., Chen H. (2024). Energy Environ. Sci..

[cit42] Zheng X., Liu W., Wei N., Zhang A., Ran G., Shan H., Huo H., Liu Y., Lu H., Xu X., Tang Z., Zhang W., Bo Z. (2023). Aggregate.

[cit43] Wang X., Zeng R., Lu H., Ran G., Zhang A., Chen Y. N., Liu Y., Liu F., Zhang W., Tang Z., Bo Z. (2023). Chin. J. Chem..

[cit44] Xing Z., Wu X., Chen T., Ye S., Wang S., Pan Y., Li S., Shi M., Chen H. (2024). J. Mater. Chem. A.

[cit45] Shen S., Liu W., Lu H., Zhang W., Zhao F., Hu B., Suo Z., Zhao K., Deng J., Mi Y., Yuan S., Ma Z., Chen Y., Liu Y., Ma Z., Lu G., Wan X., Bo Z., Song J. (2025). Adv. Funct. Mater..

[cit46] Bai Y., Xie L., Lin Z., Ai Q., Zhao F., He D. (2025). Phys. Chem. Chem. Phys..

[cit47] Kurpiers J., Neher D. (2016). Sci. Rep..

[cit48] Kniepert J., Lange I., van der Kaap N. J., Koster L. J. A., Neher D. (2014). Adv. Energy Mater..

[cit49] Li Y., Zhang Y., Zuo X., Lin Y. (2021). Chem. Commun..

[cit50] Riley D. B., Meredith P., Armin A., Sandberg O. J. (2022). J. Phys. Chem. Lett..

[cit51] Wu Y., Li Y., van der Zee B., Liu W., Markina A., Fan H., Yang H., Cui C., Li Y., Blom P. W. M., Andrienko D., Wetzelaer G. A. H. (2023). Sci. Rep..

[cit52] Sun B., Tokmoldin N., Alqahtani O., Patterson A., De Castro C. S. P., Riley D. B., Pranav M., Armin A., Laquai F., Collins B. A., Neher D., Shoaee S. (2023). Adv. Energy Mater..

[cit53] Zuo G., Shoaee S., Kemerink M., Neher D. (2021). Phys. Rev. Appl..

[cit54] Melianas A., Pranculis V., Xia Y., Felekidis N., Inganäs O., Gulbinas V., Kemerink M. (2017). Adv. Energy Mater..

[cit55] Neher D., Kniepert J., Elimelech A., Koster L. J. (2016). Sci. Rep..

[cit56] Xie M., Shi Y., Zhu L., Zhang J., Cheng Q., Zhang H., Yan Y., Zhu M., Zhou H., Lu K., Wei Z. (2023). Energy Environ. Sci..

[cit57] Zou Y., Chen H., Bi X., Xu X., Wang H., Lin M., Ma Z., Zhang M., Li C., Wan X., Long G., Zhaoyang Y., Chen Y. (2022). Energy Environ. Sci..

[cit58] Pranav M., Hultzsch T., Musiienko A., Sun B., Shukla A., Jaiser F., Shoaee S., Neher D. (2023). APL Mater..

[cit59] Nakano K., Chen Y., Xiao B., Han W., Huang J., Yoshida H., Zhou E., Tajima K. (2019). Nat. Commun..

[cit60] Pranav M., Shukla A., Moser D., Rumeney J., Liu W., Wang R., Sun B., Smeets S., Tokmoldin N., Cao Y., He G., Beitz T., Jaiser F., Hultzsch T., Shoaee S., Maes W., Luer L., Brabec C., Vandewal K., Andrienko D., Ludwigs S., Neher D. (2024). Energy Environ. Sci..

[cit61] Eisner F. D., Azzouzi M., Fei Z., Hou X., Anthopoulos T. D., Dennis T. J. S., Heeney M., Nelson J. (2019). J. Am. Chem. Soc..

[cit62] Qian D., Pratik S. M., Liu Q., Dong Y., Zhang R., Yu J., Gasparini N., Wu J., Zhang T., Coropceanu V., Guo X., Zhang M., Bredas J. L., Gao F., Durrant J. R. (2023). Adv. Energy Mater..

[cit63] Karuthedath S., Gorenflot J., Firdaus Y., Chaturvedi N., De Castro C. S. P., Harrison G. T., Khan J. I., Markina A., Balawi A. H., Pena T. A. D., Liu W., Liang R. Z., Sharma A., Paleti S. H. K., Zhang W., Lin Y., Alarousu E., Anjum D. H., Beaujuge P. M., De Wolf S., McCulloch I., Anthopoulos T. D., Baran D., Andrienko D., Laquai F. (2021). Nat. Mater..

[cit64] Zhou G., Zhang M., Chen Z., Zhang J., Zhan L., Li S., Zhu L., Wang Z., Zhu X., Chen H., Wang L., Liu F., Zhu H. (2021). ACS Energy Lett..

[cit65] Classen A., Chochos C. L., Lüer L., Gregoriou V. G., Wortmann J., Osvet A., Forberich K., McCulloch I., Heumüller T., Brabec C. J. (2020). Nat. Energy.

[cit66] Gorenflot J., Alsufyani W., Alqurashi M., Paleti S. H. K., Baran D., Laquai F. (2023). Adv. Mater. Interfaces.

[cit67] Khan J. I., Alamoudi M. A., Chaturvedi N., Ashraf R. S., Nabi M. N., Markina A., Liu W., Dela Peña T. A., Zhang W., Alévêque O., Harrison G. T., Alsufyani W., Levillain E., De Wolf S., Andrienko D., McCulloch I., Laquai F. (2021). Adv. Energy Mater..

[cit68] Qian D., Zheng Z., Yao H., Tress W., Hopper T. R., Chen S., Li S., Liu J., Chen S., Zhang J., Liu X. K., Gao B., Ouyang L., Jin Y., Pozina G., Buyanova I. A., Chen W. M., Inganas O., Coropceanu V., Bredas J. L., Yan H., Hou J., Zhang F., Bakulin A. A., Gao F. (2018). Nat. Mater..

[cit69] Benduhn J., Tvingstedt K., Piersimoni F., Ullbrich S., Fan Y., Tropiano M., McGarry K. A., Zeika O., Riede M. K., Douglas C. J., Barlow S., Marder S. R., Neher D., Spoltore D., Vandewal K. (2017). Nat. Energy.

[cit70] Vandewal K., Tvingstedt K., Gadisa A., Inganäs O., Manca J. V. (2010). Phys. Rev. B:Condens. Matter Mater. Phys..

[cit71] Vandewal K., Tvingstedt K., Gadisa A., Inganas O., Manca J. V. (2009). Nat. Mater..

[cit72] Tuladhar S. M., Azzouzi M., Delval F., Yao J., Guilbert A. A. Y., Kirchartz T., Montcada N. F., Dominguez R., Langa F., Palomares E., Nelson J. (2016). ACS Energy Lett..

[cit73] Zhu L., Tu Z., Yi Y., Wei Z. (2019). J. Phys. Chem. Lett..

[cit74] Sandberg O. J., Armin A. (2024). PRX Energy.

[cit75] Shoaee S., Stolterfoht M., Neher D. (2018). Adv. Energy Mater..

