# Supplementary material for: Balancing driving force, charge transport, and non-radiative recombination in organic solar cells with non-fused ring acceptors
Source: EES Solar. 2025 Nov 10;2(1):198–210. doi: 10.1039/d5el00136f (PMC12648669; doi:10.1039/d5el00136f)
Supplement: EL-002-D5EL00136F-s001 [file EL-002-D5EL00136F-s001.pdf]

Supplementary Information (SI) for EES Solar.
This journal is © The Royal Society of Chemistry 2025

Supplementary information

Balancing driving force, charge transport, and non-radiative recombination in organic solar cells with non-fused ring acceptors

Qian-Qian Zhang[a,b,c,d,†,‡], Manasi Pranav[e,†], De-Li Ma[a,†], Bernhard Siegmund[b,c,d], Yuyao Xu[a], Yuming Wang[b,c,d], Melissa Van Landeghem[b,c,d], Hongzheng Chen[a], Chang-Zhi Li[a,*], Dieter Neher[e,*], Koen Vandewal[b,c,d,*].

[a] State Key Laboratory of Silicon and Advanced Semiconductor Materials, Department of Polymer Science and Engineering, Zhejiang University, Hangzhou, 310027, P. R. China

[b] Hasselt University, Agoralaan 1, 3590 Diepenbeek, Belgium.

[c] IMOMEC Division, IMEC, Wetenschapspark 1, 3590 Diepenbeek, Belgium

[d] Energyville, IMO-IMOMEC, Thorpark 8320, B-3600 Genk, Belgium

[e] Institute of Physics and Astronomy, University of Potsdam, Karl-Liebknecht Straße 24/25, 14476 Potsdam, Germany

[†] These authors contributed equally to this work.

[‡] Current address: Department of Chemistry, University of Washington, Seattle, WA 98195, USA

*Corresponding author: Koen Vandewal (koen.vandewal@uhasselt.be), Dieter Neher (neher@uni-potsdam.de), Chang-Zhi Li (czli@zju.edu.cn)

1. Materials and Methods

Materials

All reagents and solvents, unless otherwise specified, were purchased from J&K Scientific, Suna Tech, Aldrich, and Energy Chemical Ltd. and were used without further purification. PM6 was purchased from Solarmer Materials Inc. The synthesis and characterization details of L0 are provided in the Supplementary information, and L2 and L4 were synthesized according to our previous work[1].

Instruments

^{1}H NMR and ^{13}C NMR spectra were recorded on a Bruker Advance III 400 (400 MHz) nuclear magnetic resonance (NMR) spectroscope. Matrix-assisted laser desorption/ionization-time of flight (MALDI-TOF) mass spectrometry (MS) spectrum were obtained on a Walters MALDI-TOF Premier mass spectrometry. UV-vis absorption spectra were measured on a Shimadzu UV-2450 spectrophotometer. Cyclic voltammetry (CV) was done on a CHI600A electrochemical workstation with Pt disk, Pt plate, and standard calomel electrode (SCE) as working electrode, a counter electrode, and reference electrode, respectively, in a 0.1 mol/L tetrabutylammonium hexafluorophosphate (Bu_4NPF_6) dichloromethane solution. The CV curves were recorded versus the potential of SCE, which was calibrated by the ferrocene-ferrocenium (Fc/Fc^+) redox couple (4.8 eV below the vacuum level). Grazing-incidence wide-angle X-ray scattering (GIWAXS) data were carried out with a Xeuss 2.0 SAXS/WAXS laboratory beamline using a Cu X-ray source (8.05 keV, 1.54 Å) and Pilatus3R 300K detector. The incidence angle is 0.2°.

Device fabrication and characterization

Organic solar cells were fabricated on glass substrates commercially pre-coated with a layer of indium tin oxide (ITO), with the conventional structure of ITO/ poly(3,4-ethylene dioxythiophene):poly(styrene-sulfonate) (PEDOT:PSS)/Active Layer/ *N,N'*-bis(3-(3-(dimethylamino)propylamino)propyl)perylene-3,4,9,10-tetracarboxylic diimide (PDINN) /Ag. Before fabrication, the substrates were cleaned consecutively by an ultrasonic bath of detergent, deionized water, acetone, and isopropanol alcohol and then treated in an ultraviolet ozone generator for 30 min. The PEDOT:PSS solution was spin-coated on the treated ITO glass at 4500 rpm for 20 s, and then annealed at 150 °C for 15 min. The photoactive layer (concentration: 15.6 mg ml^{-1}, PM6:LX = 1:1.2 and PM6:LX = 1:1.2, 0.5% vol DIO) was deposited onto the PEDOT:PSS layer by spin-coating at 3600 rpm for 40 s and thermally annealed at 90 °C for 10 min in nitrogen-filled glovebox. The PDINN was spin-coated onto photoactive layer at 3500 rpm for 40 s. Finally, Ag (100 nm) was deposited on the top through a shadow mask by thermal evaporation (1×10^{-6} mbar). The geometrical overlap of the ITO and Ag layers defines a photo-active device area of 4 mm^2.

J-V curves were recorded in nitrogen atmosphere under an AM 1.5G solar simulator (Newport 91195A) with an intensity equivalent to 100 mW cm^{-2}, using a Keithley 2400 source meter. The EQE spectrum for each device was measured under chopped (135 Hz) monochromatic illumination from a Xe lamp (100 W, Newport) modulated by monochromator (Cornerstone 130, Newport) and an optical wheel chopper.

Space charge limited current (SCLC)

The SCLC measurements were performed using the following device structures:

- Hole-only devices: ITO/PEDOT:PSS /blend film/MoO_3/Ag
- Electron-only devices: ITO/ZnO/blend film/PDINN/Ag

The mobilities were extracted using the Mott-Gurney law:

$$J = \frac{9}{8}\varepsilon_r \varepsilon_0 \mu \frac{V^2}{L^3}$$

where ε_r ≈3 is the dielectric constant, ε_0 is the permittivity of free space, μ is the carrier mobility, L≈100 nm is the film thickness, and V is the applied voltage.

Bias-assisted charge extraction (BACE)

In the BACE measurement, a studied device was held under illumination of a continuous wave LED (520 nm, InsaneWare) at open circuit (using an Agilent 81150A pulse generator) to create a stable photogenerated excess carrier density. The laser diode was operated at 500 Hz with a duty cycle of 50%, such that illumination lasted 1 ms and the diode was switched off for also 1 ms. As soon as the illumination was switched off, a high reverse bias was applied to the cell terminals by the same fast pulse generator (Agilent 81150A), and the respective extraction current transient due to the photogenerated charges was measured using a Keysight Infiniium 9000 Series Agilent DSO9104H oscilloscope via a 10 Ω resistor. The displacement charge was taken into account via a measurement with the same voltage jump performed in the dark.

Time-delayed collection field (TDCF)

In TDCF, the device was excited with a laser pulse from a diode-pumped, Q-switched Nd:YAG laser (NT242, EKSPLA) with ~6 ns pulse duration at a typical repetition rate of 500 Hz. An Agilent 81150A pulse generator was used to apply a square voltage transient waveform constituting the pre-bias V_{pre} and collection bias V_{coll}. To compensate for the internal latency of the pulse generator, the laser pulse was delayed and homogeneously scattered in an 85 m long silica fiber (LEONI) after triggering a photodiode. The device was illuminated while held at different pre-bias V_{pre}. After a pre-set delay time (calculated from the falling slope of the photodiode trigger), a high reverse bias V_{coll} was applied to extract all the charges generated in the device. V_{pre} and V_{coll} were sent by the Agilent 81150A pulse generator through a home-built current amplifier, which was triggered by a fast photodiode (EOT, ET2030TTL). The current flowing through the device was measured via a 10 Ω resistor in series with the sample and recorded with an oscilloscope (Agilent DSO9104H). To avoid non-geminate recombination of photogenerated free charge carriers prior to extraction, the intensity of light is kept very low and the delay time of collection is set to ~1ns.

Modified TDCF

Since the range of the pre-bias sweep during generation extends up to open-circuit conditions where there is a significant density of injected charge carriers from the electrodes, we verify the field dependence of generation using modified TDCF (mTDCF). For mTDCF, the same experimental setup as classical TDCF was used. The difference in pre-biasing conditions was achieved by programming a square-type waveform which was then fed into the Agilent 81150A pulse generator. Therein, the delay parameters for the V_{pre} and V_{coll} voltage steps were pre-defined prior to the measurement, such that the V_{pre} was applied to the device for a duration equaling the 3xRC time of the device plus the laser slew rate. As in classical TDCF, the laser beam incidence is delayed to compensate for the internal latency of the function generator.

Resistive photovoltage (RPV)

The laser pulse to generate charges comes from a diode-pumped, Q-switched Nd:YAG laser (NT242, EKSPLA) with 5 ns pulse duration at a repetition rate of 50 Hz. Photovoltage transients were recorded with an oscilloscope (Agilent

DSO9104H) with a load resistance of 1M Ohm. Low laser pulse fluences were used to prevent screening of the internal field and a built-up of charges inside the device. The transit times of slow and fast charge carriers were fit the RPV transient with the function described in **Figure 3c**. We assume that the transport of charge carriers as mobile charges causes 95% of the RPV transient and the prolonged saturation at longer timescales, accounting for the remaining 5% of the total change in electrostatic potential, is due to time-dependent de-trapping and subsequent transport of slow charge carriers lying in lower energy levels in the density of states. (Further details will be published in upcoming works.) The intersection of the tangent of the intermediate voltage rise with that of the maximum voltage drop was used to ascertain the slower carrier transit time.

Bias-dependent photoluminescence (PL) and absolute PL (PLQY)

Bias-dependent PL measurements were performed using a 520 nm CW laser diode (Insaneware) for steady-state illumination, and the intensity of the laser was adjusted to a 1 sun equivalent by illuminating the solar cell under short-circuit (provided by a Keithley 2400) and matching the current density reading to the J_{SC} obtained in the sun simulator. The excitation beam was focused onto the sample using a stage of mirrors and lenses. Bias voltages ranging from open-circuit voltage to -4 V were applied to the sample using the same Keithley 2400. To ensure that only the active layer is illuminated and contributes to the emission response, we masked the measured pixels. The emission spectra were recorded with an Andor Solis SR393i-B spectrograph with a silicon DU420A-BR-DD detector and an Indium Gallium Arsenide DU491A-1.7 detector. A calibrated Oriel 63355 lamp was used to correct the spectral response. PL spectra were recorded with different gratings at central wavelengths of 800, 1100, and 1400 nm, and merged afterwards. For PLQY measurements, the same laser was used but the excitation beam was channeled through an optical fiber into an integrating sphere containing the sample. A second optical fiber was used from the output of the integrating sphere to the Andor Solis SR393i-B spectrograph. The spectral photon density was obtained from the corrected detector signal (spectral irradiance) by division through the photon energy ($h\nu$), and the photon numbers were calculated from the numerical integration, using a Matlab code.

Fourier-transform photocurrent spectroscopy (FTPS-EQE) and electroluminescence quantum yield (EQE_{EL})

FTPS-EQE measurements were performed using an INVENIOR (Bruker Optics) with an external detector. A low-noise current amplifier was used to amplify the photocurrent generated under illumination of the devices, with the illumination light modulated by the Fourier-transform infrared setup. The EQE_{EL} was recorded with a home-built setup using a 100 mm^2 Hamamatsu silicon photodiode, which was placed directly in front of the device with at a distance of 12 mm. A Keithley 2400 source meter was used to supply voltage and record the injected current, and simultaneously another Keithley 2400 was used to measure the emitted photons from the device. The radiance of the OLEDs was estimated using the same EQE_{EL} setup and Lambertian emission was assumed. The thickness of the glass substrate was considered when calculating the solid angle.

Electroluminescence (EL) measurements

The EL emission spectra were recorded using an Andor spectrometer (Kymera 328i-D2-SIL) with an InGaAs array detector (DU490A-1.7), which was cooled to -60 ℃. An 830 nm long-pass filter was used to exclude the presence of peaks originating from the second order of diffraction from the grating. The voltage bias was applied to devices with a Keithley 2400 external current/voltage source meter. The injected current was close to V_{OC} of OSC devices measured

under AM1.5G illumination at 100 mW cm^2. The injection-dependent EL spectra of PM6:L0 (w/o DIO) is shown in **Figure S16c** and **S16d**, indicating that the EL shape of the spectra remains unchanged when going from an injection current close to 1×J_{SC} (~0.8 mA) to 3×J_{SC} (~2.4 mA). The details of injection current density and applied voltages were displayed in **Table S8** and **S9**. The system was wavelength-calibrated by an argon lamp (HG-2, Ocean Optics) with a resolution better than 0.5 nm and irradiance-calibrated by a National Institute of Standards and Technology (NIST) calibrated light source (Ava-Light-HAL-CAL-Mini, 300–2,500 nm). To recover the high-energy tail part of the EL spectra, a CCD silicon array detector (DU416A-LDC-DD) with a 590-nm long-pass filter was used.

Temperature-dependent EL measurements

For temperature-dependent EL measurements, regular EL measurements were conducted on the sample placed inside a cryostat. The temperature within the cryostat was controlled with liquid nitrogen and a LakeShore (Model 335) cryogenic temperature controller, ranging from 295K down to ca. 220K. The injected current using a Keithley 2400 SMU was kept constant for each temperature, and was calculated from the J_{SC} at 1 sun.

2. Synthesis of L0

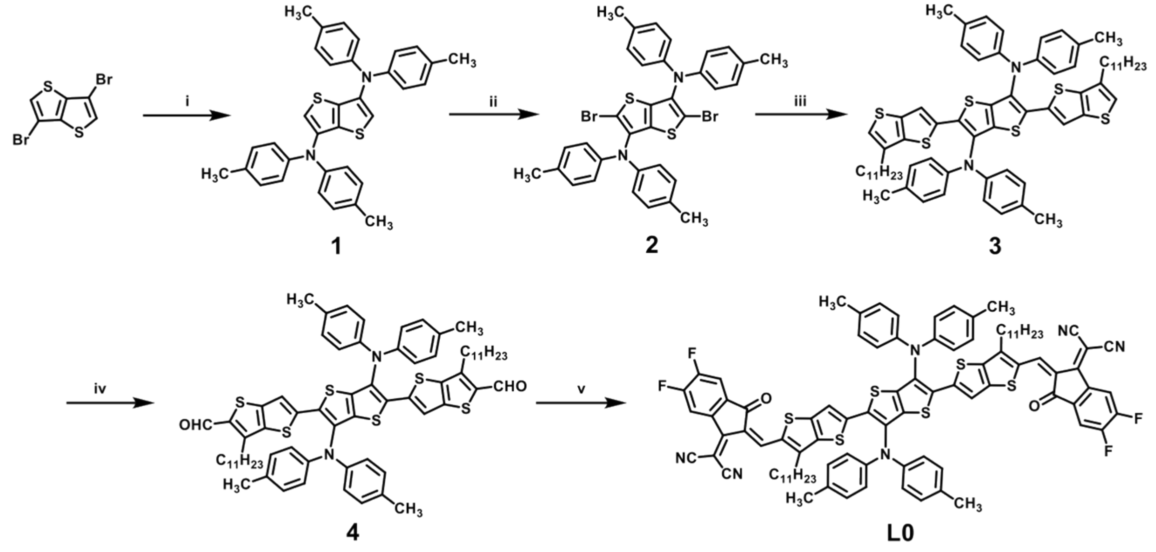

Scheme S1. The synthetic routes of L0.

*N*³,*N*³,*N*⁶,*N*⁶-tetra-p-tolylthieno[3,2-*b*]thiophene-3,6-diamine (**1**)

A mixture of 3,6-dibromothieno[3,2-*b*]thiophene (400 mg, 1.35 mmol), Bis(4-methylphenyl)amine (863 mg, 3.62 mmol), $Pd_2(dba)_3$ (62 mg, 0.067 mmol), NaO*t*-Bu (271 mg, 2.82 mmol), $(t\text{-}Bu)_3PBF_4$ (155 mg, 0.534 mmol) and o-xylene (20 mL) was added to a flame-dried and nitrogen-filled bottom flask (100 mL). The mixture was followed by three successive freeze-pump-thaw cycles. After back fill with nitrogen gas, the mixture was refluxed for 48 h. Followed by rotary evaporation, the crude product was dispersed with dichloromethane and filtered through a Buchner funnel to collect the filtrate. After removing the solvent, silica gel column chromatography was used to purify the product with the mixture of petroleum ether and dichloromethane (4:1, v/v) as the eluent, yielding a compound **1** as pale yellow solid (452 mg, 55 %).

^{1}H NMR (400 MHz, $CDCl_3$, δ/ppm) 7.08 (d, J = 8.4 Hz, 8H), 7.02 (d, J = 8.5 Hz, 10H), 2.32 (s, 12H).

2,5-Dibromo-*N*³,*N*³,*N*⁶,*N*⁶-tetra-p-tolylthieno[3,2-*b*]thiophene-3,6-diamine (**2**)

NBS (0.276 mg, 1.55 mmol) in 10 mL of dichloromethane was added dropwise to a solution of compound 1 (400 mg, 0.7 mmol) in DCM at 0 °C in the dark. The reaction mixture was stirred at room temperature for 12 hours. After filtration, the organic solvent was removed under reduced pressure. The crude product was purified by flash column chromatography on silica gel eluting with dichloromethane/petroleum ether (1:4, v/v) to give compound **2** (490 mg, 96% yield) as an off-white powder.

^{1}H NMR (400 MHz, $CDCl_3$, δ/ppm) 7.07 (dd, *J* = 8.8, 2.9 Hz, 8H), 6.96 - 6.89 (m, 8H), 2.32 (s, 13H).

$N^{3'}$,$N^{3'}$,$N^{6'}$,$N^{6'}$-tetra-*p*-tolyl-6,6''-diundecyl-[2,2':5',2''-terthieno[3,2-*b*]thiophene]-3',6'-diamine (**3**)

Compound **2** (400 mg, 0.52 mmol), trimethyl(6-undecylthieno[3,2-*b*]thiophen-2-yl)stannane (593 mg, 1.3 mmol) and $Pd(PPh_3)_4$ (12 mg, 0.02 mmol), together with toluene (30 mL) were added to a flame-dried and nitrogen-filled bottom flask (100 mL). The mixture was followed by three successive freeze-pump-thaw cycles. After being filled back with nitrogen gas, the mixture was refluxed for 24 h. Following rotary evaporation, the crude product was dissolved in

dichloromethane, and washed with water. The organic phase was collected and dried with anhydrous $MgSO_4$. The solvent was filtered through a Buchner funnel and the filtrate was collected. The dichloromethane was removed by rotary evaporation, and the crude product was dissolved in 50 mL of n-hexane solution. After filtering through diatomaceous earth, the solution was concentrated to 20 mL by heating. The concentrated solution was allowed to stand overnight at -20 °C to precipitate a solid, which was filtered to obtain a crude intermediate **3** as a yellow solid (500 mg, yield 78%), which was used directly in the next step without purification.

3',6'-Bis(di-p-tolylamino)-6,6''-diundecyl-[2,2':5',2''-terthieno[3,2-*b*]thiophene]-5,5''-dicarbaldehyde (**4**)

$POCl_3$ (0.4 mL) was added to 0.5 mL DMF under nitrogen at 0°C. After stirring for 1 hour, compound **3** (400 mg, 0.33 mmol) dissolved in 1,2-dichloroethane (10 mL) was added dropwise to the resulting Vilsmeier reagent. The mixture was stirred at 0°C for 30 minutes and then heated to 85°C for 24 hours. After cooling to room temperature, saturated sodium bicarbonate solution was added and stirred for 3 hours. The mixture was extracted three times with dichloromethane; the combined organic phases were washed with saturated saline solution, dried over anhydrous $MgSO_4$, filtered and concentrated under reduced pressure. The residue was chromatographed on a silica gel column eluting with dichloromethane /petroleum ether (1:1, v/v) to give compound **4** (314 mg,75%) as an orange solid.

^{1}H NMR (400 MHz, $CDCl_3$, δ/ppm) 9.98 (s, 2H), 7.19 (s, 2H), 7.05 (q, *J* = 8.6 Hz, 16H), 2.96 (t, *J* = 7.5 Hz, 4H), 2.29 (s, 12H), 1.64 (s, 4H), 1.26 (s, 33H), 0.87 (d, *J* = 7.1 Hz, 7H).

2,2'-((2*Z*,2'*Z*)-((3',6'-bis(di-*p*-tolylamino)-6,6''-diundecyl-[2,2':5',2''-terthieno[3,2-*b*]thiophene]-5,5''-diyl)bis(methaneylylidene))bis(5,6-difluoro-3-oxo-2,3-dihydro-1*H*-indene-2,1-diylidene))dimalononitrile (**L0**)

A mixture of compound **4** (300 mg, 0.24 mmol) and 2-(5,6-difluoro-3-oxo-2,3-dihydro-1*H*-inden-1-ylidene) malononitrile (220 mg, 0.96 mmol) in chloroform (20 mL) was degassed, and then pyridine (0.5 mL) was added. The mixture was stirred at reflux under nitrogen overnight and the solvent was removed under reduced pressure. The crude product was purified on silica gel chromatography using dichloromethane/petroleum ether (1:1, v/v) as eluent to give the target compound **L0** (330 mg, 83%) as a black solid.

^{1}H NMR (400 MHz, $CDCl_3$, δ/ppm) 8.99 (s, 1H), 8.53 (dd, *J* = 10.0, 6.5 Hz, 1H), 7.65 (t, *J* = 7.6 Hz, 1H), 7.24 (s, 1H), 7.10 (d, *J* = 8.3 Hz, 4H), 7.07 - 7.03 (m, 4H), 3.00 (t, *J* = 7.7 Hz, 2H), 2.31 (s, 6H), 1.73 - 1.57 (m, 2H), 1.24 (s, 18H), 0.92 - 0.83 (m, 4H).

^{13}C NMR (126 MHz, $CDCl_3$, δ/ppm) 185.93, 159.18, 155.53, 155.42, 153.47, 153.44, 153.36, 153.33, 152.46, 151.50, 145.41, 142.84, 142.35, 136.65, 136.63, 136.59, 136.56, 136.49, 135.70, 135.50, 134.85, 134.65, 134.60, 134.59, 122.18, 120.38, 117.00, 115.03, 114.86, 114.69, 112.72, 112.57, 77.41, 77.16, 76.91, 68.81, 32.05, 30.73, 30.03, 29.81, 29.74, 29.67, 29.58, 29.49, 22.83, 21.02, 14.27.

MS (MALDI-TOF): Calcd. for $C_{94}H_{78}F_4N_4O_2S_6$ (M+): 1596.03, Found: 1595.26.

3. Supplementary Note

The fill factor (FF) was calculated from the carrier mobilities and the bimolecular recombination constant following the analytical model developed by Neher et al.. [2] This model introduces a figure-of-merit parameter, α, which accounts for the competition between carrier transport and recombination in low-mobility semiconductors such as organic materials.

$$\alpha^2 = \frac{qk_2d^3J_G}{4\mu_{fast}\mu_{slow}(k_BT)^2} = \frac{qk_2d^3Gd^4}{4\mu_{fast}\mu_{slow}(k_BT)^2} \quad \textbf{(1)}$$

Where q is the elementary charge, k_2 is the bimolecular recombination constant (from TDCF/BACE measurements), d is the active layer thickness, J_G is the photogenerated current density, μ_{fast} and μ_{slow} are the faster and slower carrier mobilities (from RPV measurements), k_B is the Boltzmann constant, and T is the temperature.

The α parameter is then used to calculate the normalized open-circuit voltage u_{OC} and subsequently the analytical FF for different $V_{OC:}$

$$u_{OC} = \frac{qV_{OC}}{(1+\alpha)k_BT} \quad \textbf{(2)}$$

$$FF = \frac{u_{OC} - \ln(0.79 + 0.66u_{OC}^{1.2})}{u_{OC}+1} \quad \textbf{(3)}$$

The calculation details for voltage loss (**Equation 4**):

$$\begin{aligned} q\Delta V_{loss} &= E_{gap} - qV_{OC} \quad \textbf{(4)} \\ &= \left(E_{gap} - qV_{OC}^{SQ}\right) + \left(qV_{OC}^{SQ} - qV_{OC}^{rad}\right) + \left(qV_{OC}^{rad} - qV_{OC}\right) \\ &= \left(E_{gap} - qV_{OC}^{SQ}\right) + q\Delta V_{OC}^{rad,\ below\ gap} + q\Delta V_{OC}^{non-rad} \\ &= \Delta V_1 + \Delta V_2 + \Delta V_3 \end{aligned}$$

Radiative Recombination Above the Gap ($q\Delta V_1$): In an ideal device operating within the Shockley Queisser limit, the maximum voltage (qV_{OC}^{SQ}) is reduced by $q\Delta V_1$ relative to Eg due to radiative recombination from absorption above the optical gap ($q\Delta V_1 = E\text{g} - qV_{OC}^{SQ}$). The calculation of qV_{OC}^{SQ} accords to **Equation 5**

$$qV_{OC}^{SQ} = kT \times ln\left(\frac{J_{SC,SQ}}{J_{0,SQ}} + 1\right) = kT \times ln\left(\frac{q\int_{Eg}^{\infty} \emptyset_{AM1.5}(E)dE}{q\int_{Eg}^{\infty} \emptyset_{bb}(E)dE} + 1\right) \quad \textbf{(5)}$$

where k is the Boltzmann constant, T is the absolute temperature, $\emptyset_{AM1.5}$ is the solar radiation photon flux, $\emptyset_{bb}$ is a black body radiation at 300 K.

Radiative Recombination Below the Gap ($q\Delta V_2$): This component ($q\Delta V_2 = q\Delta V_{OC}^{rad,\ below\ gap}$) arises from extra radiative recombination due to sub-gap absorption, primarily associated with charge transfer (CT) state absorption linked to ΔE_{offset} in organic solar cells (OSCs). The difference between $V_{OC}^{SQ}$and V_{OC}^{rad} is caused by the non-stepfunction like absorptance (or EQE_{PV}) of the real-world devices:

$$qV_{OC}^{rad} = kT \times ln\left(\frac{J_{SC,rad}}{J_{0,rad}} + 1\right) = kT \times ln\left(\frac{q\int_{Eg}^{\infty} EQE_{PV}\emptyset_{AM1.5}(E)dE}{q\int_{Eg}^{\infty} EQE_{PV}\emptyset_{bb}(E)dE} + 1\right) \quad \textbf{(6)}$$

Nonradiative Recombination ($q\Delta V_3$): The third loss term ($q\Delta V_3$ = $q\Delta V_{OC}^{non-rad}$ = $-kT$ × ln(EQE_{EL})) is due to nonradiative recombination, where EQE_{EL} denotes the electroluminescence quantum efficiency of the solar cell under dark conditions with injected charge carriers.

4. Supplementary Figures

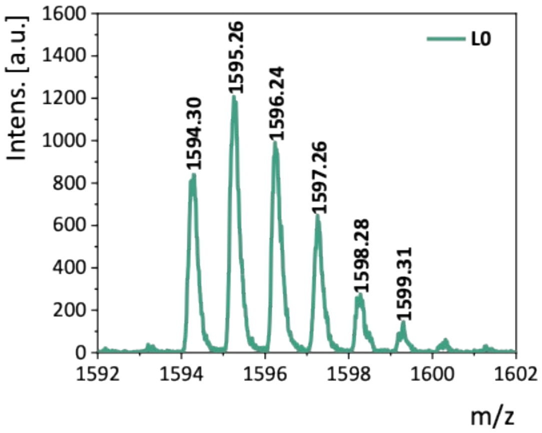

Figure S1. The matrix-assisted laser desorption-time of flight mass spectrometry of L0.

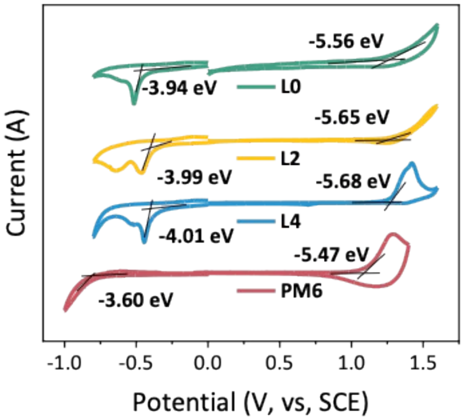

Figure S2. The cyclic voltammogram curves for LX and PM7.

Table S1 The optoelectronic properties of LX.

Acceptor	λ_{max}^{film} [eV]	λ_{max}^{sol} [eV]	Red shift [eV]	λ_{edge}^{film} [eV]	E_g^{opt} [eV]	$E_{HOMO/LUMO}$ [eV]	E_g^{CV} [eV]
L0	1.63	1.71	0.08	1.39	1.48	-5.56/-3.94	1.62
L2	1.64	1.76	0.12	1.40	1.52	-5.65/-3.99	1.66
L4	1.66	1.82	0.16	1.43	1.56	-5.68/-4.01	1.67

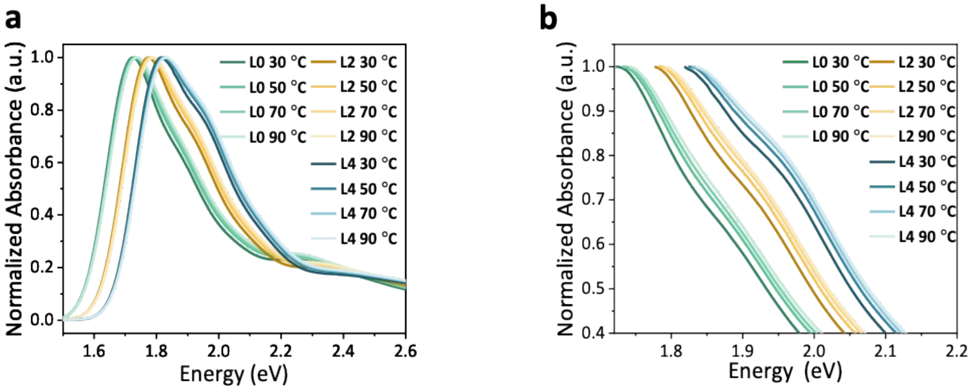

Figure S3. Temperature-dependent absorption spectra of LX acceptors (L0, L2, and L4) in chlorobenzene solution measured at 30, 50, 70, and 90 °C. (a) Normalized absorption spectra over the full range. (b) Enlarged view of the 1.75–2.2 eV region: L0 exhibits a more pronounced spectral shift compared to L2 and L4.

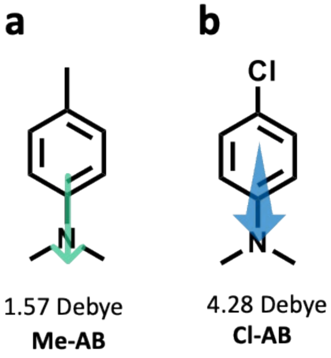

Figure S4. Dipole moments of the template molecules representing (a) methyl-substituted side chain (Me-AB) and (b) chlorine-substituted side chain (Cl-AB). The increase in dipole moment from Me-AB (1.57 Debye) to Cl-AB (4.28 Debye) arises from the high electronegativity of chlorine.

Table S2 The summary of photovoltaic parameters for organic solar cells based on the non-fused ring acceptors with the same backbone as L series acceptors.

Active layer	J_{SC} [mA cm^{-2}]	V_{OC} [V]	FF	PCE	Ref
D18:2BTh-2F-C_2	24.53	0.915	0.70	15.59	[3]
D18:OC4-4Cl-C8	24.40	0.90	0.75	16.56	[4]
D18:3TT-C2-F	24.13	0.90	0.79	17.19	[5]
D18:3TT-C2-Cl	23.14	0.89	0.78	16.17	[5]
D18:3TT-C2	22.31	0.90	0.77	15.42	[5]
D18:BM-2F	24.21	0.91	0.73	16.15	[6]
PM6:BM-2F	21.92	0.91	0.71	14.09	[6]
D18:2BTh-2F	23.61	0.90	0.72	15.44	[7]
PBDB-T:2BTh-2F	24.02	0.90	0.72	14.53	[7]
D18-DPA-3	22.15	0.92	0.71	14.62	[8]
D18-DPA-3	24.41	0.93	0.74	16.67	[8]
D18-DPA-3	22.54	0.92	0.73	15.12	[8]
D18:3TTS-4F	24.17	0.92	0.72	15.86	[9]
D18:3TTB-4F	24.83	0.95	0.74	17.38	[9]
D18:3TTDPA	21.97	0.94	0.67	13.85	[10]
PM6:L1	23.00	0.85	0.79	15.4	[1]
PM6:L2	23.86	0.89	0.77	16.2	[1]
PM6:L0	22.01	0.91	0.71	14.11	
PM6:L2	24.01	0.89	0.77	16.45	
PM6:L4	23.00	0.84	0.79	15.39	

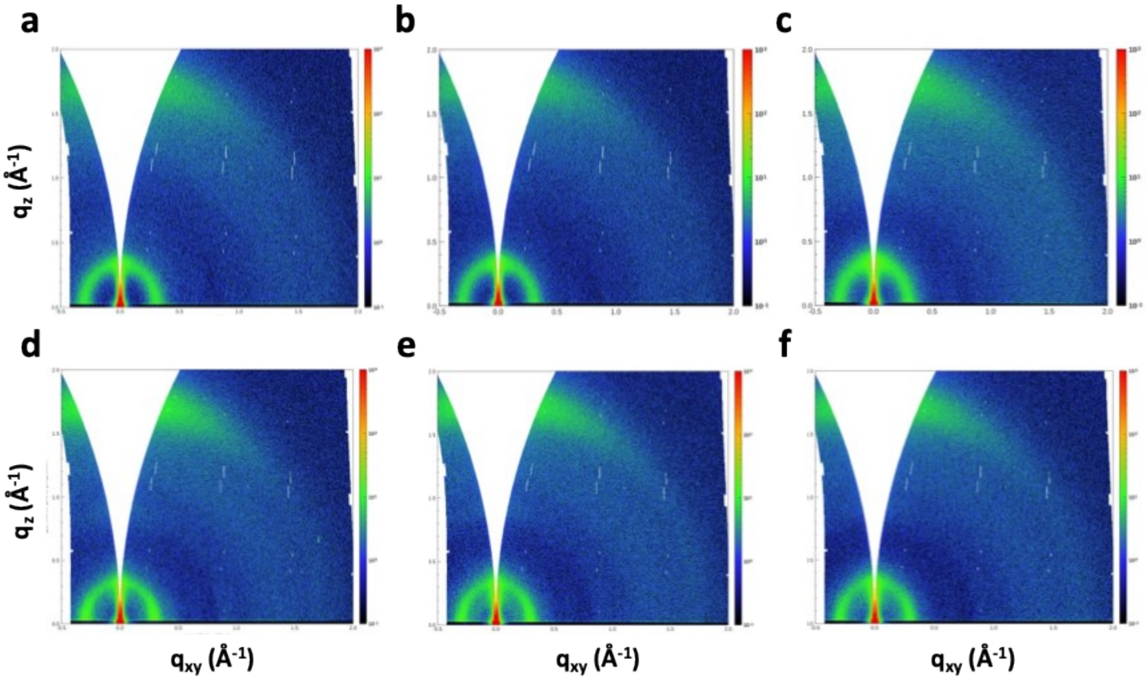

Figure S5. Two dimensional grazing incidence wide-angle X-ray scattering (2D-GIWAXS) images of (a) PM6:L0 (w/o DIO), (b) PM6:L2 (w/o DIO), (c) PM6:L4 (w/o DIO), (d) PM6:L0 (w/ DIO), (e) PM6:L2 (w/ DIO), (f) PM6: L4 (w/ DIO) films.

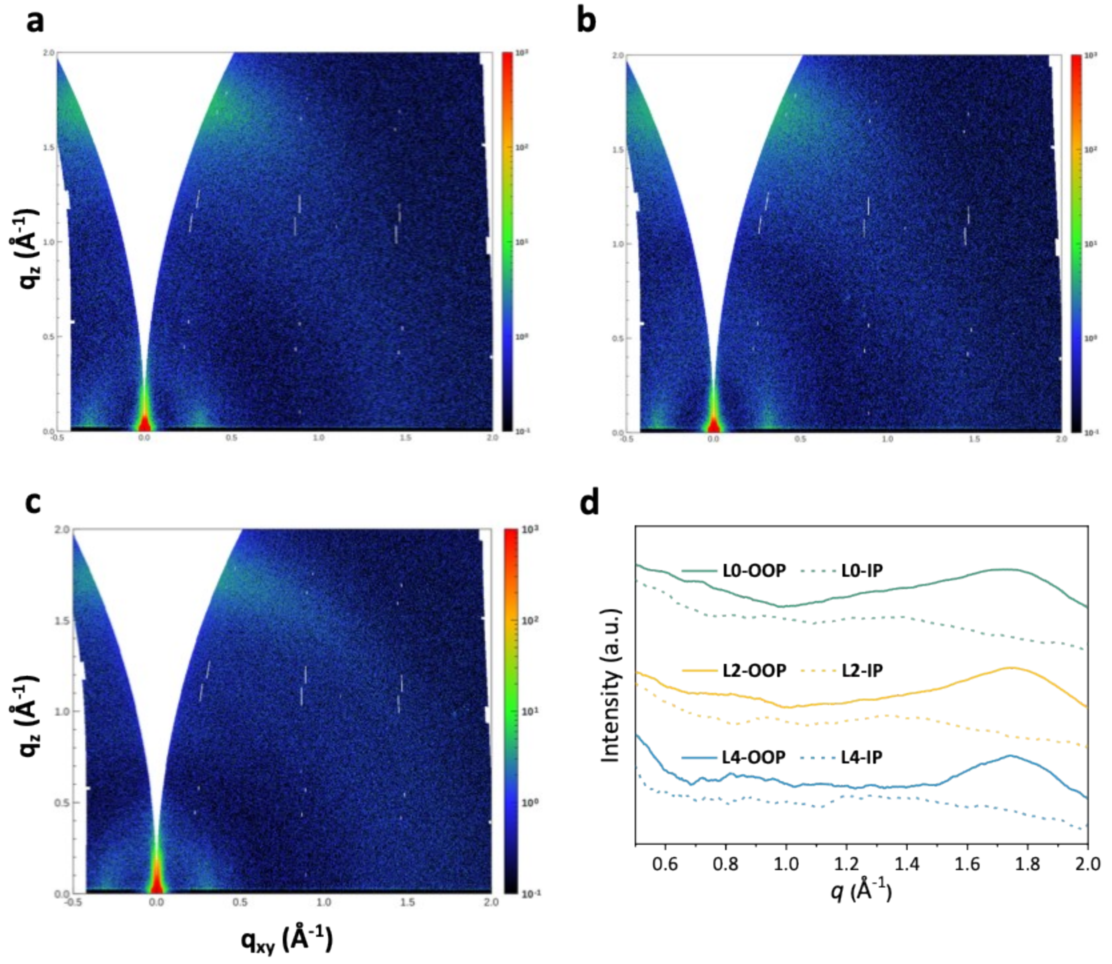

Figure S6. 2D-GIWAXS images of (a) L0, (b) L2, (c) L4 neat films. (d) The GIWAXS intensity curves for LX neat films (solid line, OOP direction; dash line, IP direction).

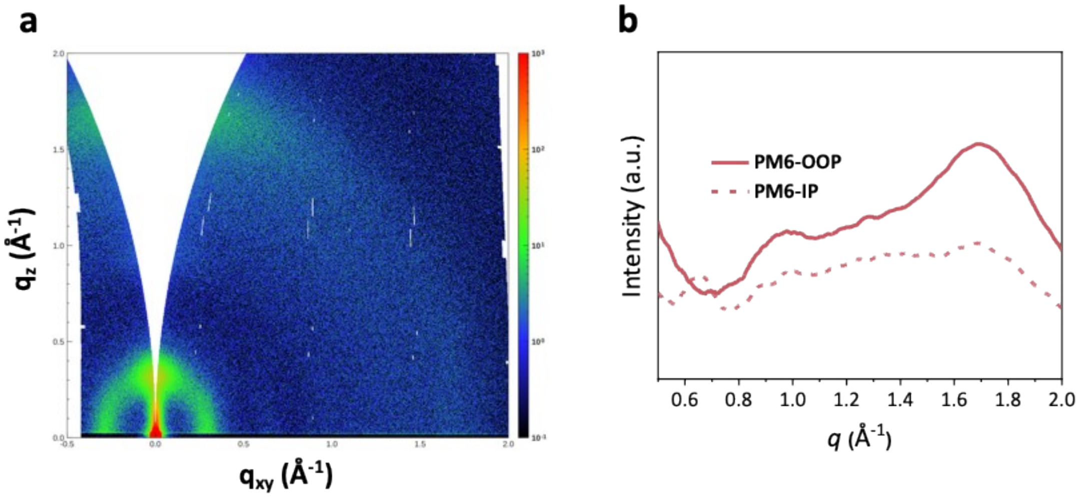

Figure S7. (a) 2D-GIWAXS images of PM6 neat films. (b) GIWAXS intensity curves for PM6 neat films (solid line, OOP direction; dash line, IP direction).

Table S3. Detailed Data of GIWAXS Characterization.

		PM6:L0 (w/o DIO)	PM6:L2 (w/o DIO)	PM6:L4 (w/o DIO)	PM6:L0 (w/ DIO)	PM6:L2 (w/ DIO)	PM6:L4 (w/ DIO)
OOP-Peak1	*q* [Å^{-1}]	1.74	1.74	1.75	1.74	1.74	1.74
	d [Å]	3.61	3.61	3.59	3.61	3.61	3.62
OOP-Peak2	*q* [Å^{-1}]	/	/	0.97	0.91	0.90	0.93
	d [Å]	/	/	6.50	6.92	7.02	6.73
IP-Peak2	*q* [Å^{-1}]	0.94	0.94	0.91	0.88	0.90	0.90
	d [Å]	6.70	6.68	6.88	7.12	6.96	6.98

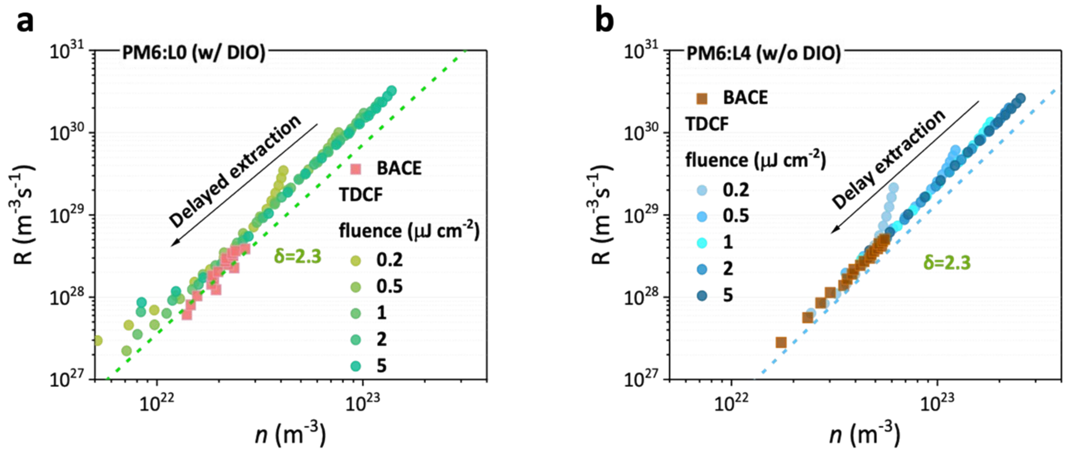

Figure S8. The recombination rate *R* versus mobile charge carrier density *n* from time-delayed collection field (TDCF) and bias-assisted charge extraction (BACE) for (a) PM6:L0 (w/ DIO) and (b) PM6:L4 (w/o DIO).

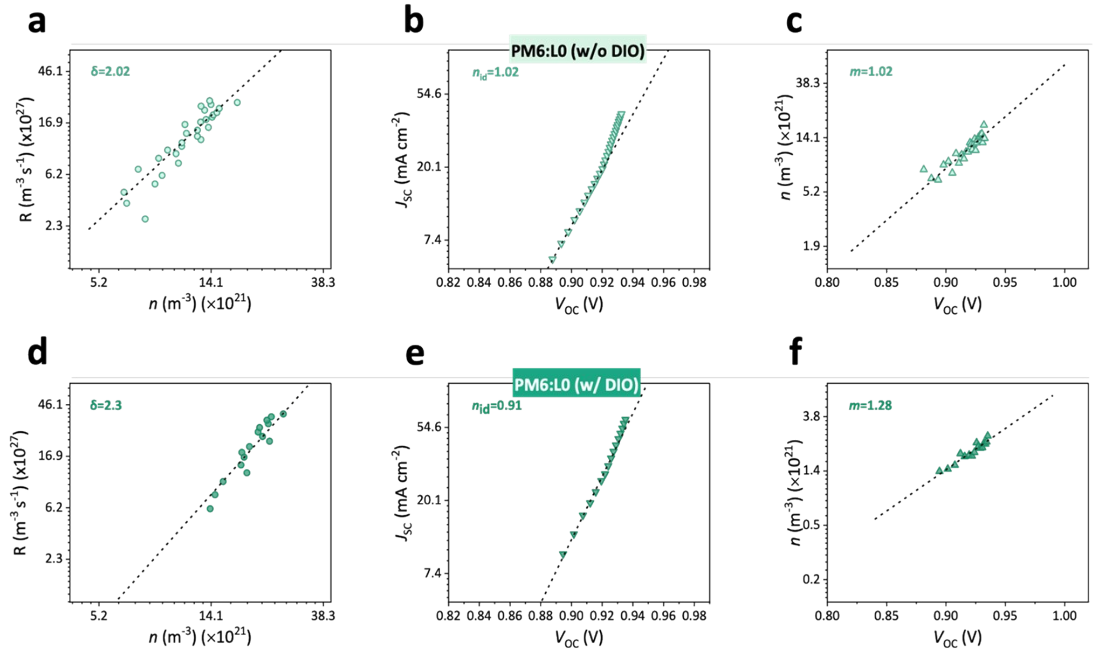

Figure S9. The recombination rate *R* versus mobile charge carrier density *n* for (a) PM6:L0 (w/o DIO) and (d) PM6:L0 (w/ DIO). The J_{SC} versus V_{OC} for (b) PM6:L0 (w/o DIO) and (e) PM6:L0 (w/ DIO). The *n* versus V_{OC} for (c) PM6:L0 (w/o DIO) and (f) PM6:L0 (w/ DIO). These data were measured from BACE.

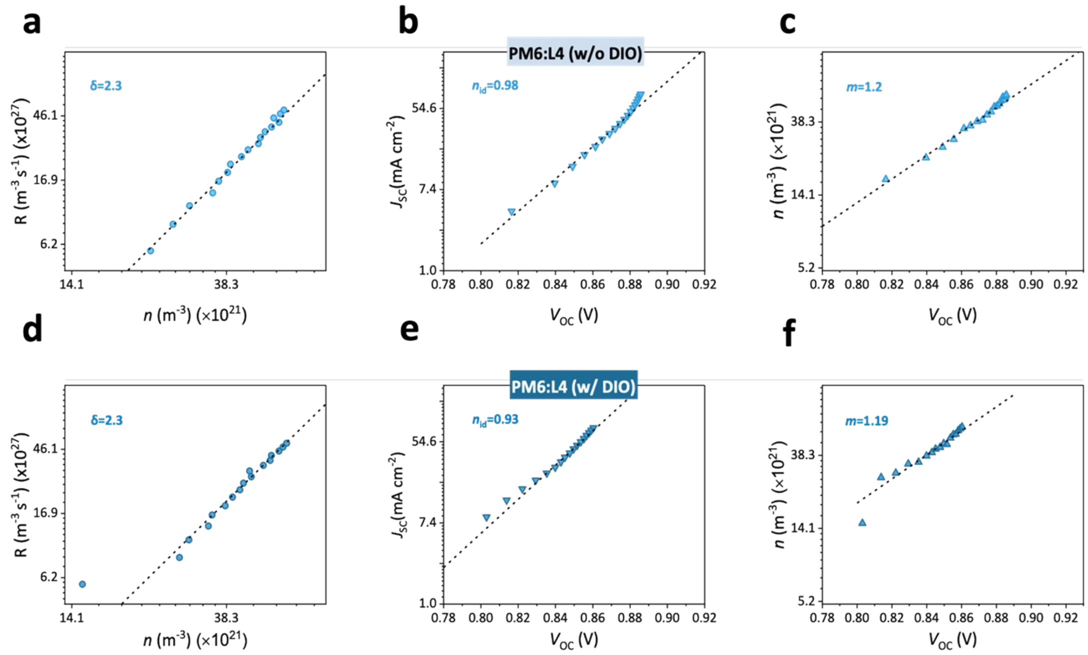

Figure S10. The recombination rate *R* versus mobile charge carrier density *n* for (a) PM6:L4 (w/o DIO) and (d) PM6:L4 (w/ DIO). The J_{SC} versus V_{OC} for (b) PM6:L4 (w/o DIO) and (e) PM6:L4 (w/ DIO). The *n* versus V_{OC} for (c) PM6:L4 (w/o DIO) and (f) PM6:L4 (w/ DIO). These data were measured from BACE.

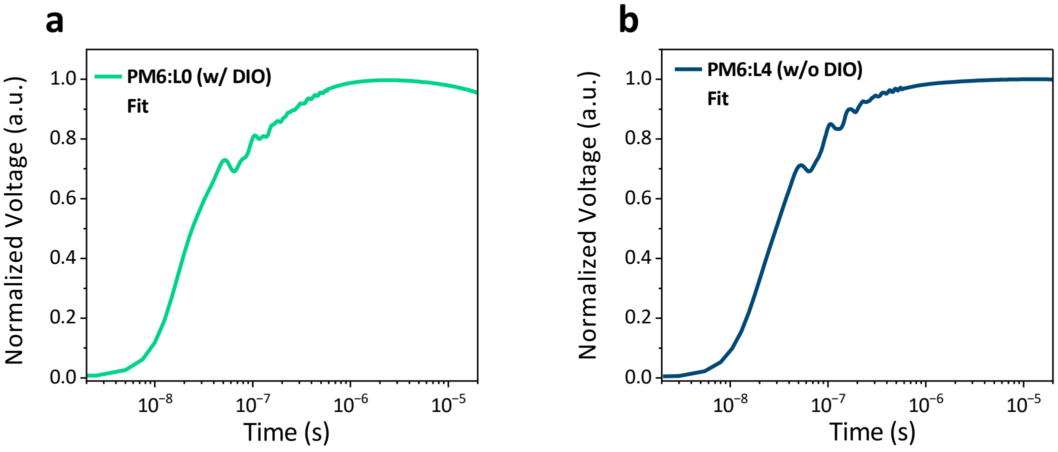

Figure S11. The RPV transients fitted with fitting functions and the transit times of carriers for (a) PM6:L0 (w/ DIO) and (b) PM6:L4 (w/o DIO). The mobilities obtained using these transit times are tabulated in **Table S4**.

Table S4 The carrier mobility of devices based on PM6:LX measured from RPV.

	μ_h [$cm^2V^{-1}s^{-1}$]	μ_e [$cm^2V^{-1}s^{-1}$]	μ_e/μ_h

PM6:L0 (w/o DIO)	1.7×10^{-4}	2.9×10^{-3}	17.05
PM6:L4 (w/o DIO)	1.4×10^{-4}	2.1×10^{-3}	15.00
PM6:L0 (w/ DIO)	2.8×10^{-4}	2.4×10^{-3}	8.57
PM6:L4 (w/ DIO)	2.7×10^{-4}	2.4×10^{-3}	8.89

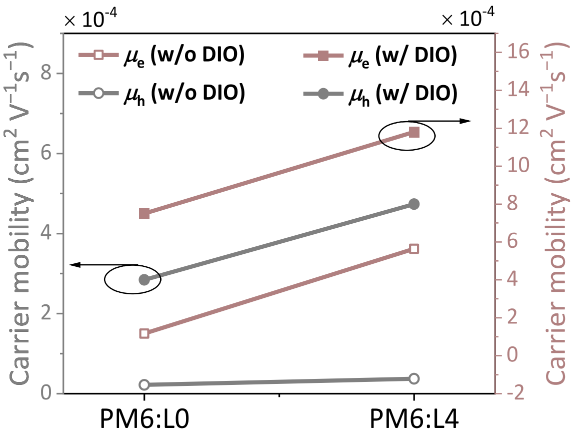

Figure S12. The carrier mobility of the hole-only and electron-only devices based on PM6:LX.

Table S5 The carrier mobility of devices based on PM6:LX measured from SCLC.

	μ_h [$cm^2V^{-1}s^{-1}$]	μ_e [$cm^2V^{-1}s^{-1}$]
PM6:L0 (w/o DIO)	2.2×10^{-5}	1.5×10^{-4}
PM6:L4 (w/o DIO)	3.7×10^{-5}	3.6×10^{-4}
PM6:L0 (w/ DIO)	4.0×10^{-4}	7.5×10^{-4}
PM6:L4 (w/ DIO)	8.0×10^{-4}	1.2×10^{-3}

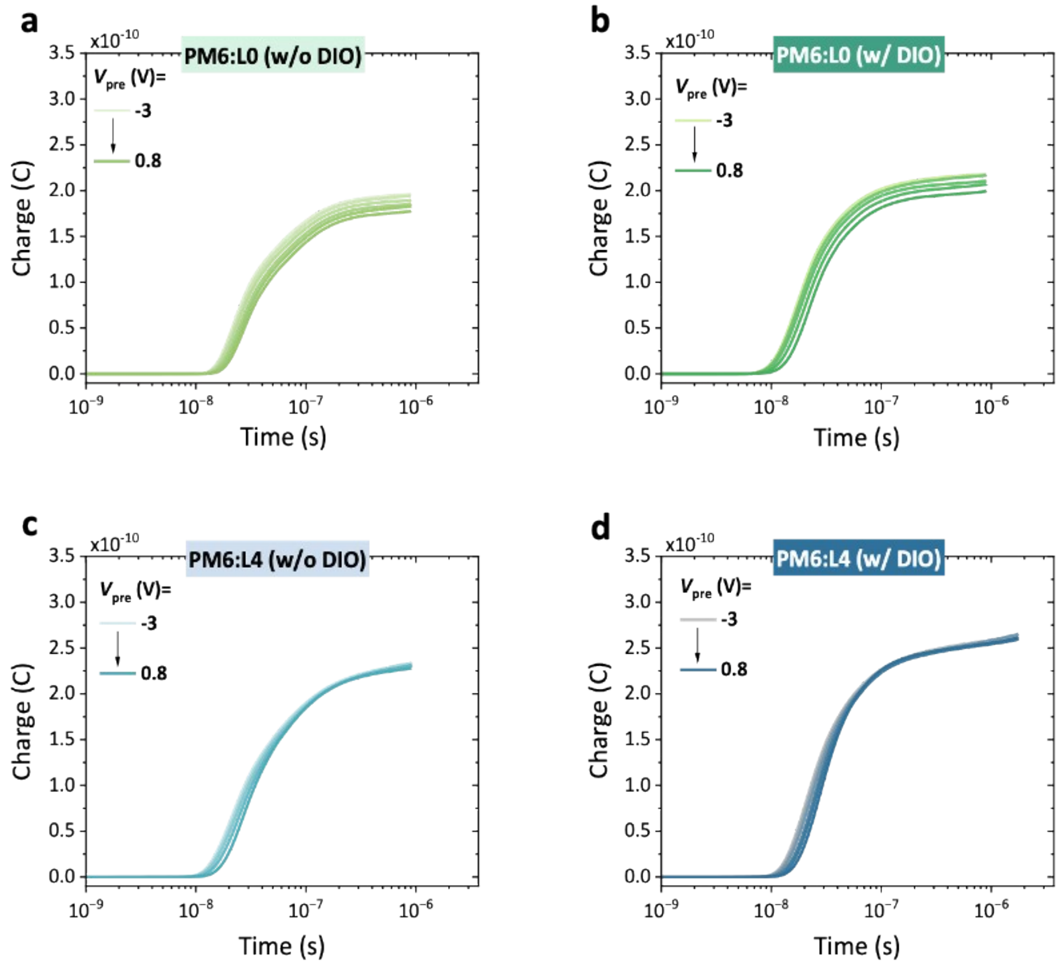

Figure S13. The integrated transient current from classical TDCF measurements, showing the collected charge as a function of the extraction time under different V_{pre} for (a) PM6:L0 (w/o DIO), (b) PM6:L0 (w/ DIO), (c) PM6:L4 (w/o DIO), and (d) PM6:L4 (w/ DIO).

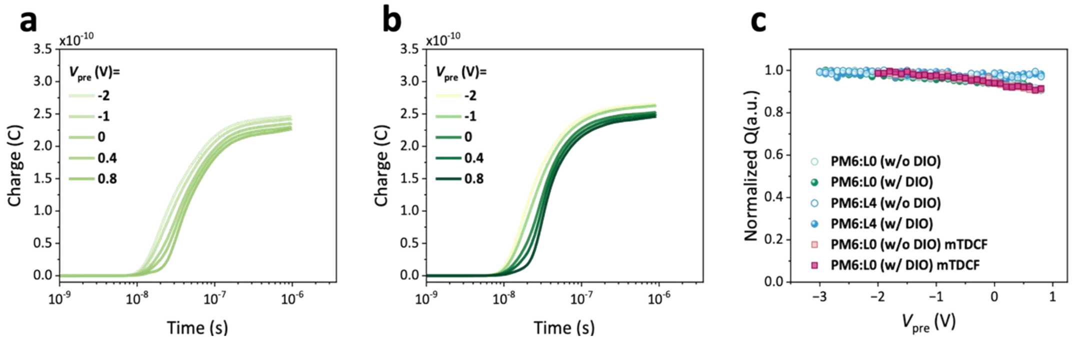

Figure S14 The integrated transient current from modified TDCF (mTDCF) measurements, showing the collected charge as a function of the delay time under different V_{pre} for (a) PM6:L0 (w/o DIO) and (b) PM6:L0 (w/ DIO). (c) Normalized Q_{gen} of L0 and L4 systems, compared with the L0 systems measured from mTDCF.

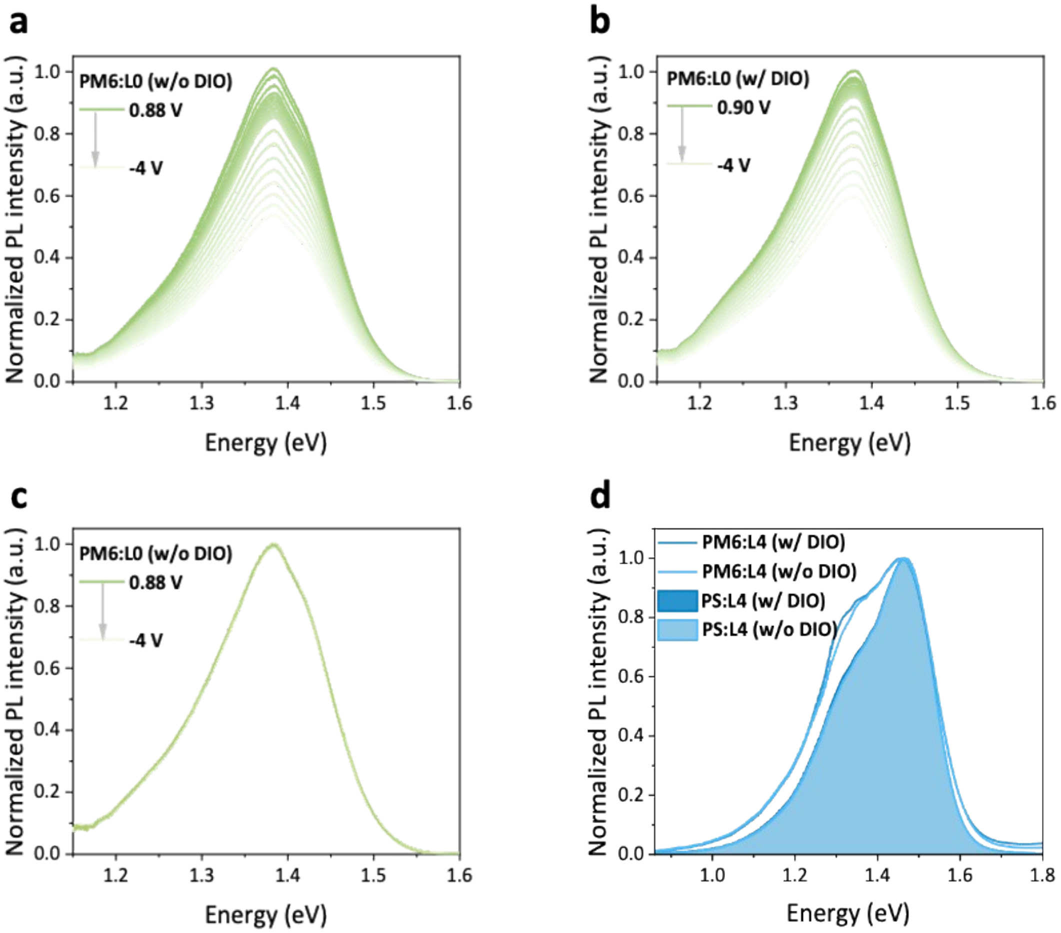

Figure S15. The photoluminescence for (a) PM6:L0 (w/o DIO), (b) PM6:L0 (w/ DIO) under different bias, with quenched density. (c) The normalized PL curves of PM6:L0 (w/o DIO) under different bias, with no change in spectra shape. (d) Normalized photoluminescence of PM6:L4 blends and polystyrene:L4 with and without DIO.

Table S5 The PLQY data of corresponding devices.

	L0 (w/o DIO]	L4 (w/o DIO]	L0 (w/ DIO]	L4 (w/ DIO]
PLQY (PM6:LX)	2.16×10^{-3}	3.03×10^{-4}	2.88×10^{-3}	3.11×10^{-4}
PLQY (PS:LX)	1.38×10^{-2}	1.86×10^{-2}	3.18×10^{-2}	1.95×10^{-2}

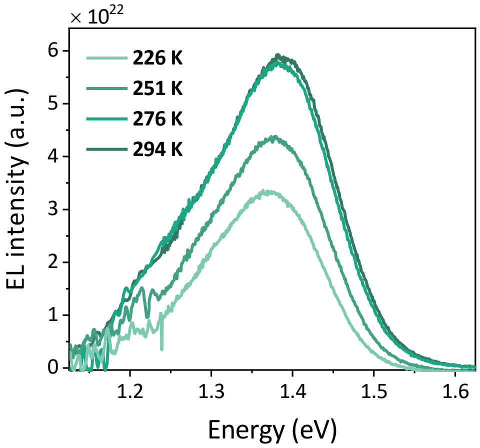

Figure S16. The EL curves of PM6:L0 (w/o DIO) under different temperature.

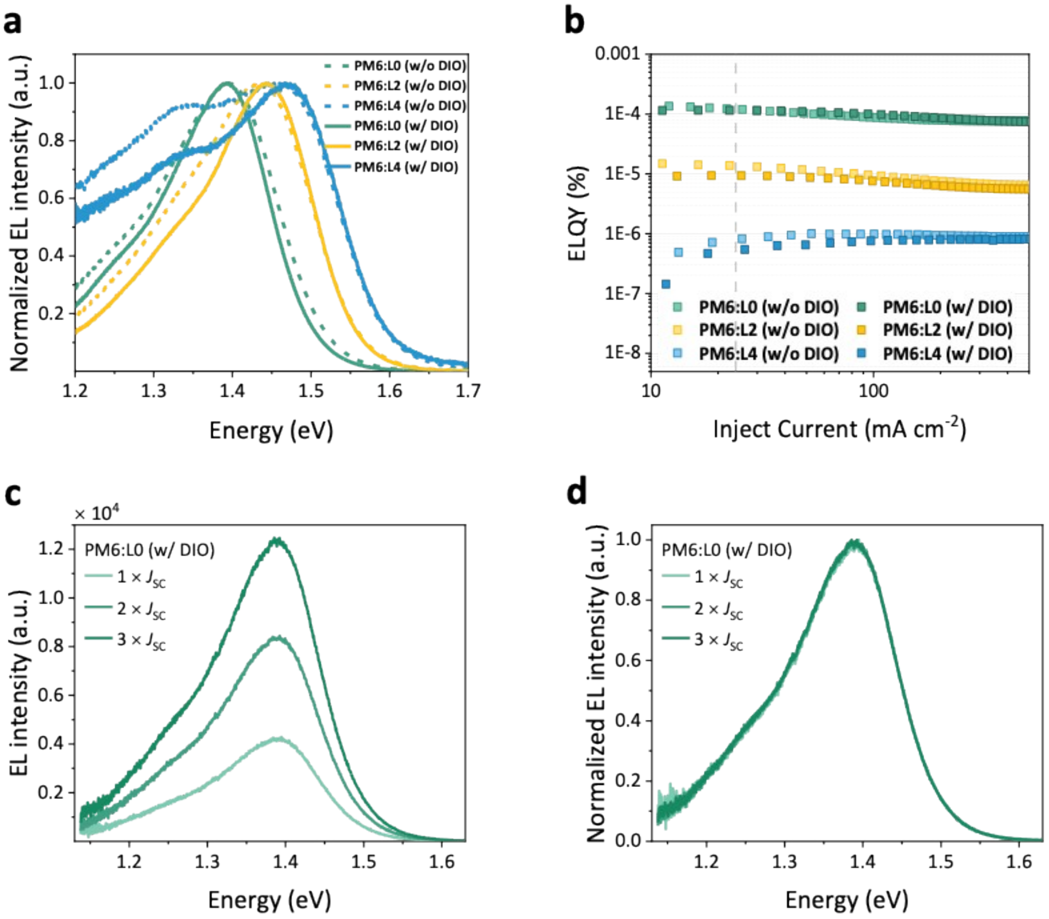

Figure S17 The (a) EL and (b) ELQY curves of the corresponding devices. (c) The EL curves of PM6:L0 (w/ DIO) under different bias showing quench EL density. (d) The normalized EL curves of PM6:L0 (w/ DIO) under different bias, with no change in spectra shape.

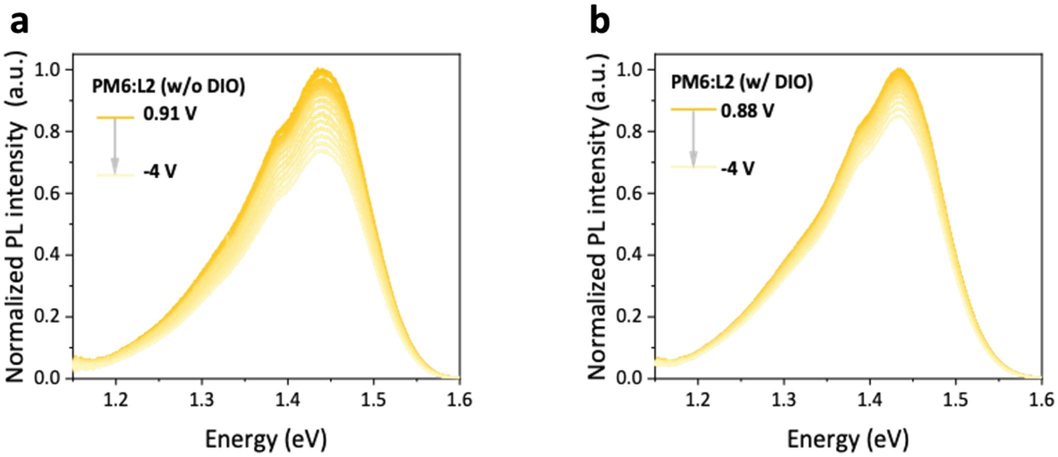

Figure S18 The photoluminescence for (a) PM6:L2 (w/o DIO), (b) PM6:L2 (w/ DIO) under different bias, with quenched density.

Table S6 The EL measurements details for the studied systems.

	PM6:L0 (w/o DIO)	PM6:L2 (w/o DIO)	PM6:L4 (w/o DIO)	PM6:L0 (w/ DIO)	PM6:L2 (w/ DIO)	PM6:L4 (w/ DIO)
Injected current [mA]	0.85	0.88	0.89	0.88	0.88	0.87
Applied voltage [V]	1.14	1.03	0.93	0.98	0.97	0.89

Table S7 The EL measurements details for PM6:L0 (w/ DIO) under different voltage.

Injected current [mA]	0.80	1.60	2.40
Applied voltage [V]	0.97	1.03	1.09

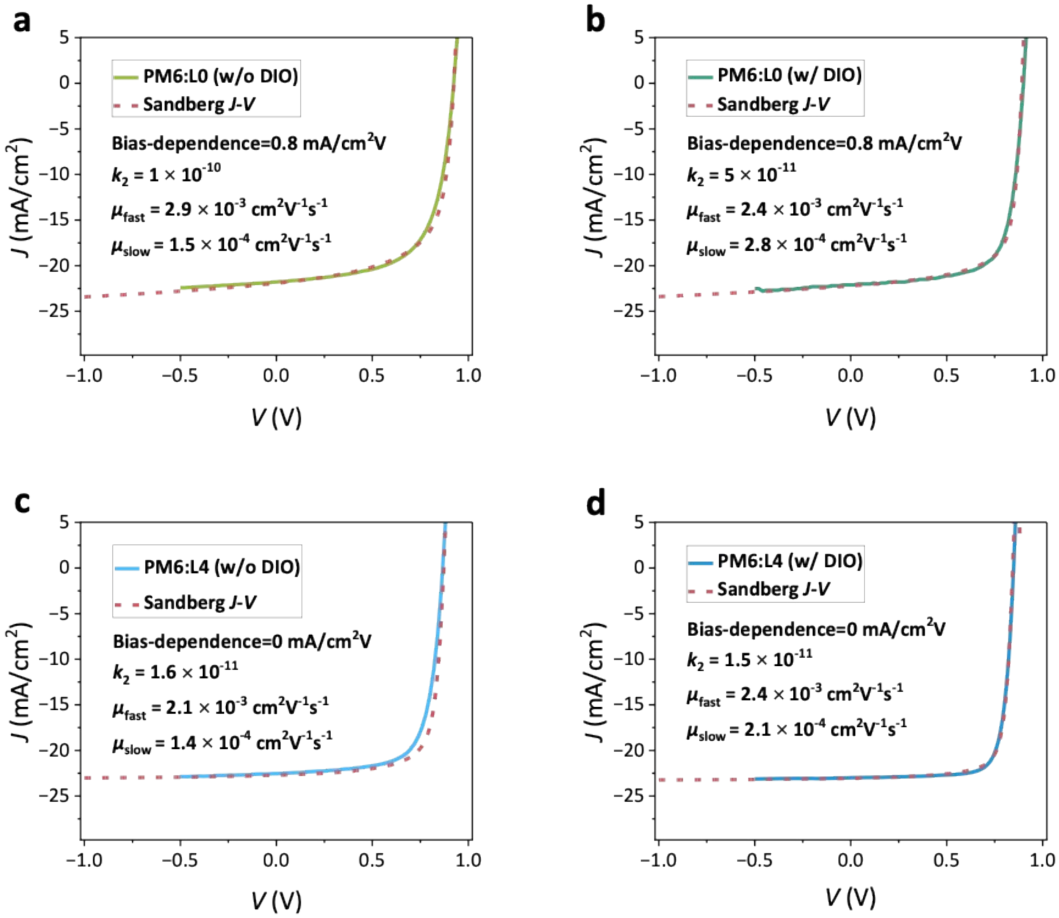

Figure S19 Experimental *J-V* curve of L0- and L4- based devices under different processing conditions, overlaid with fits from the modified diode equation of Sandberg et al.,[11] $J(V) = \frac{1}{\sqrt{1+K(V)}}\left(1 - \frac{\theta kT}{qF_0(V)d}\right)(J_{ideal}(V) + \zeta(V))$. Here ζ(V) is the field-dependent addend term expressed in mA cm^{-2}V^{-1}, obtained from TDCF measurements, and is used as an input to the model along with the bimolecular recombination coefficient (from BACE) and the charge mobilities (from RPV).

5. NMR Spectra

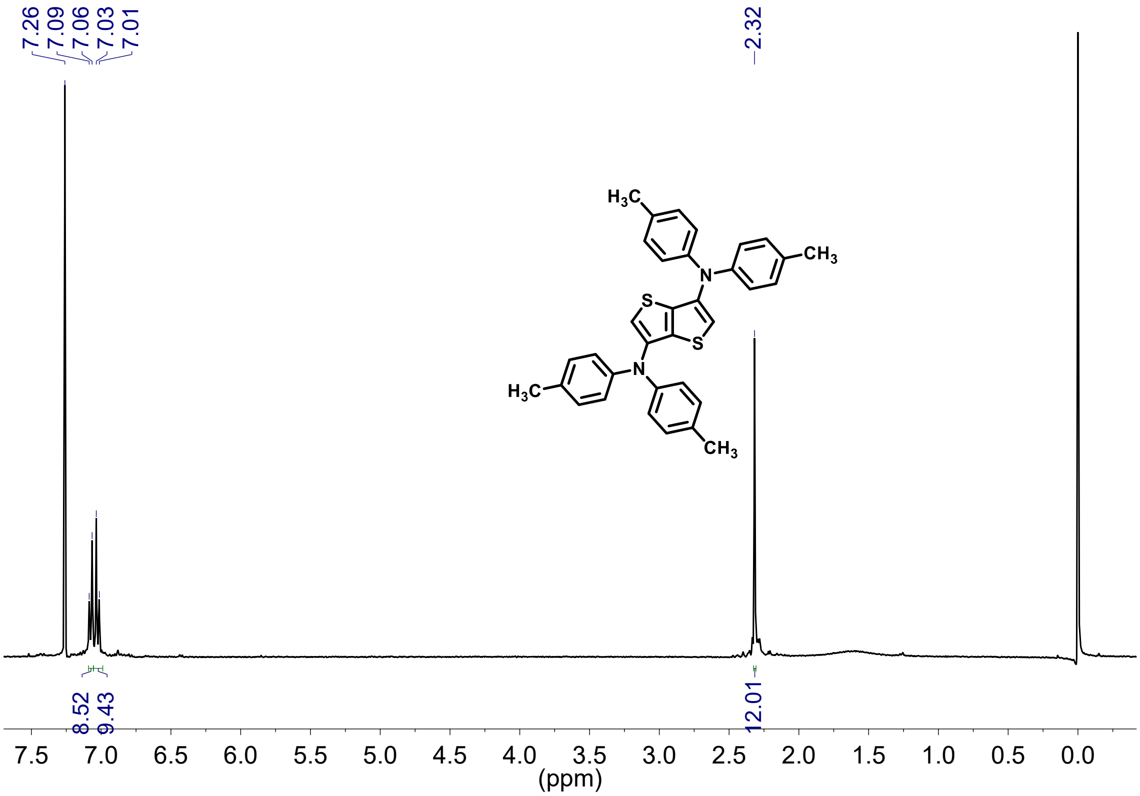

Figure S20 ^{1}H NMR spectrum of *N*^{3},*N*^{3},*N*^{6},*N*^{6}-tetra-p-tolylthieno[3,2-*b*]thiophene-3,6-diamine in $CDCl_3$ solution.

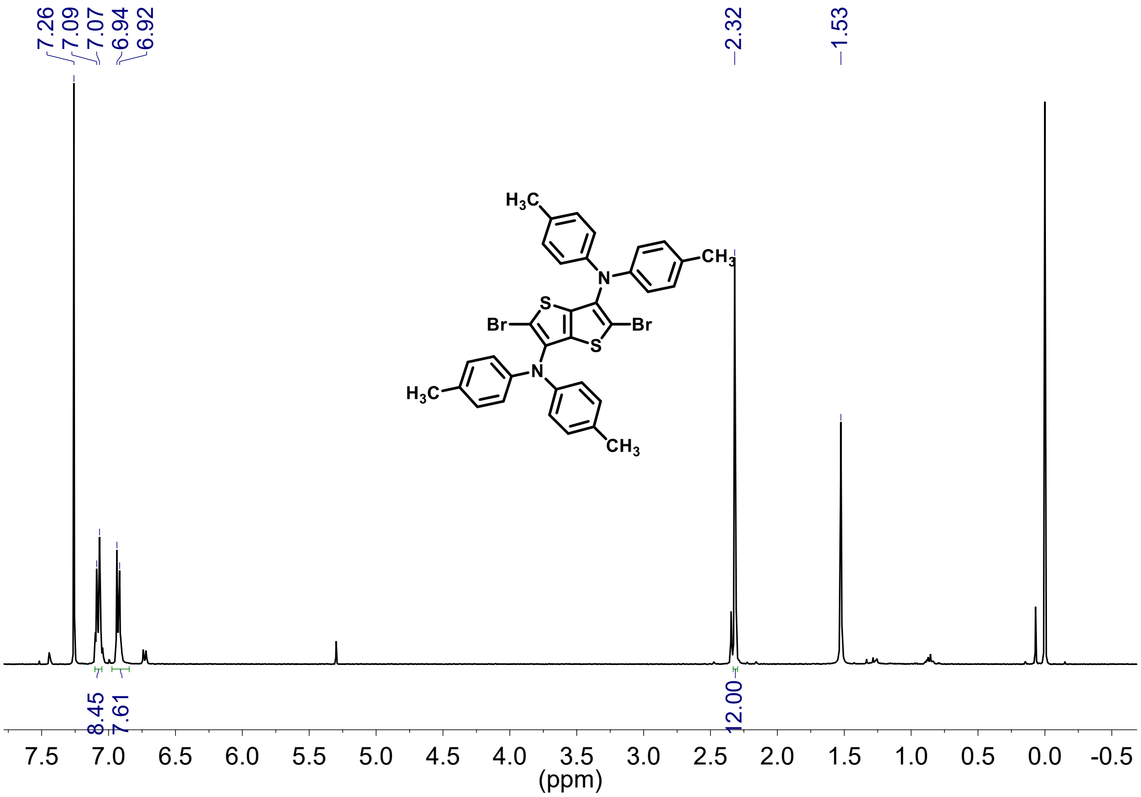

Figure S21 ^{1}H NMR spectrum of 2,5-Dibromo-*N*^{3},*N*^{3},*N*^{6},*N*^{6}-tetra-p-tolylthieno[3,2-*b*]thiophene-3,6-diamine in $CDCl_3$ solution.

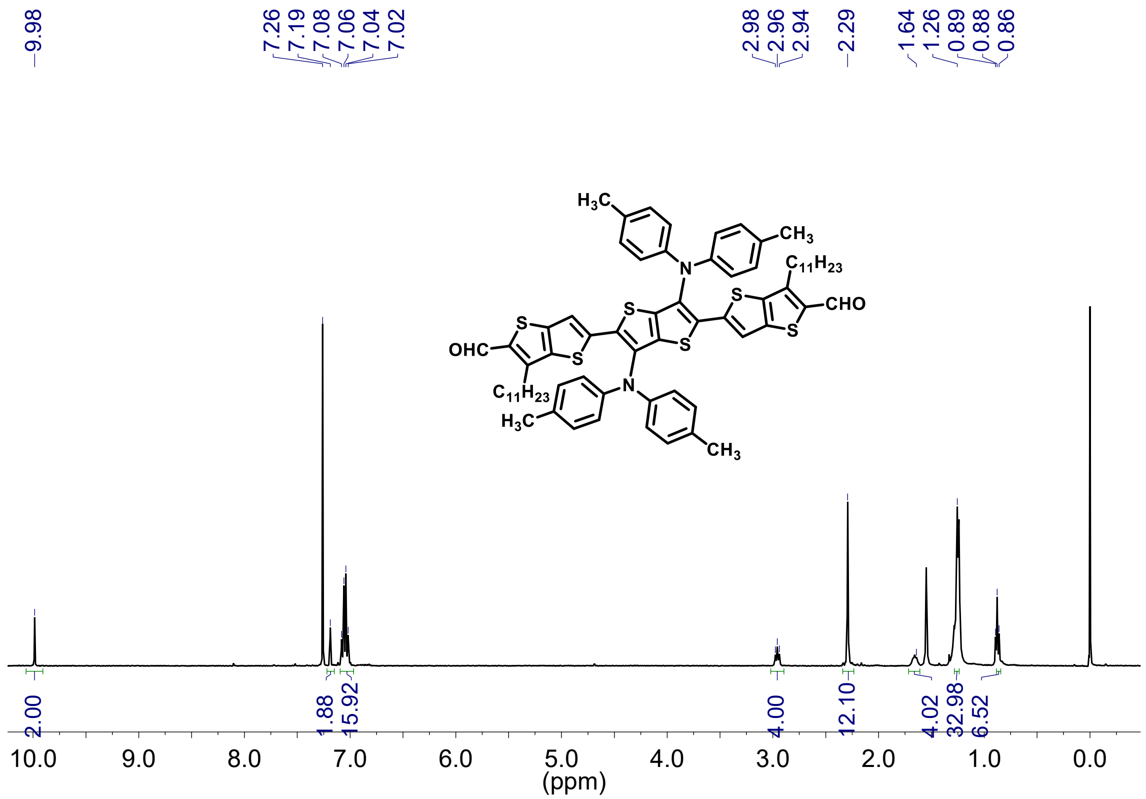

Figure S22 ^{1}H NMR spectrum of $N^{3'}$,$N^{3'}$,$N^{6'}$,$N^{6'}$-tetra-p-tolyl-6,6''-diundecyl-[2,2':5',2''-terthieno[3,2-*b*]thiophene]-3',6'-diamine in $CDCl_3$ solution.

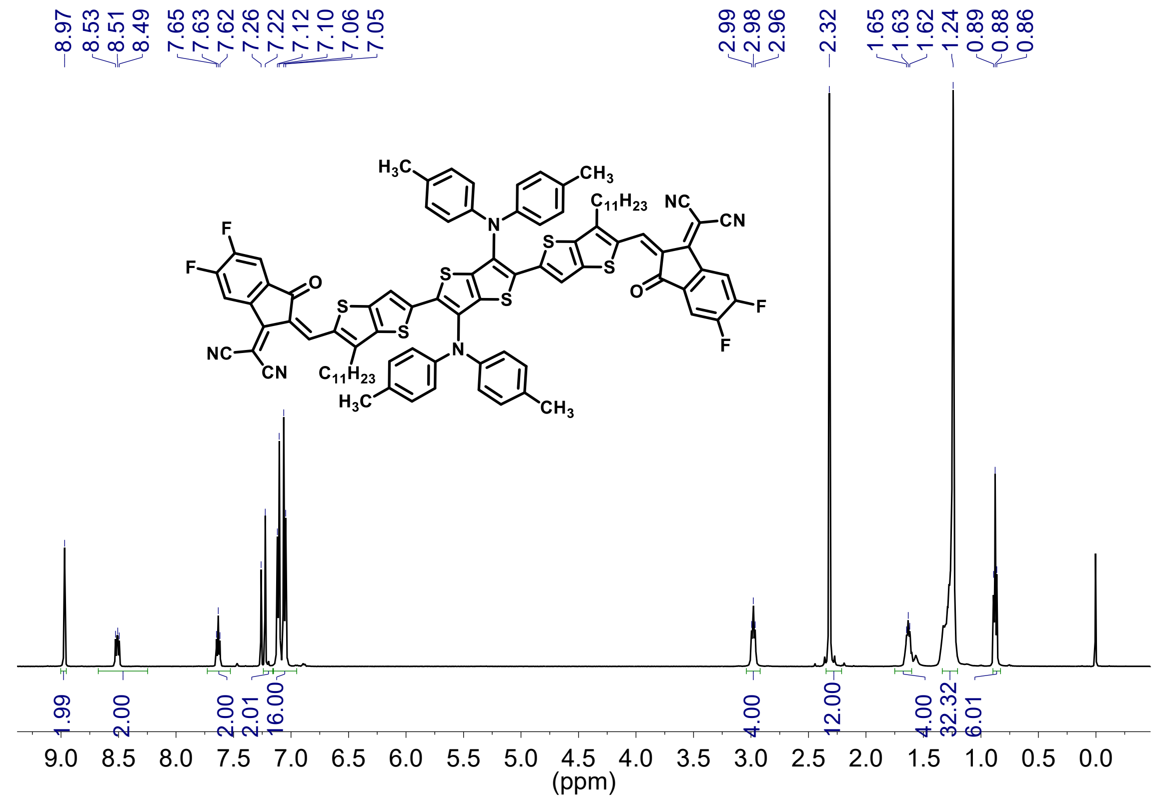

Figure S23 ^{1}H NMR spectrum of L0 in $CDCl_3$ solution.

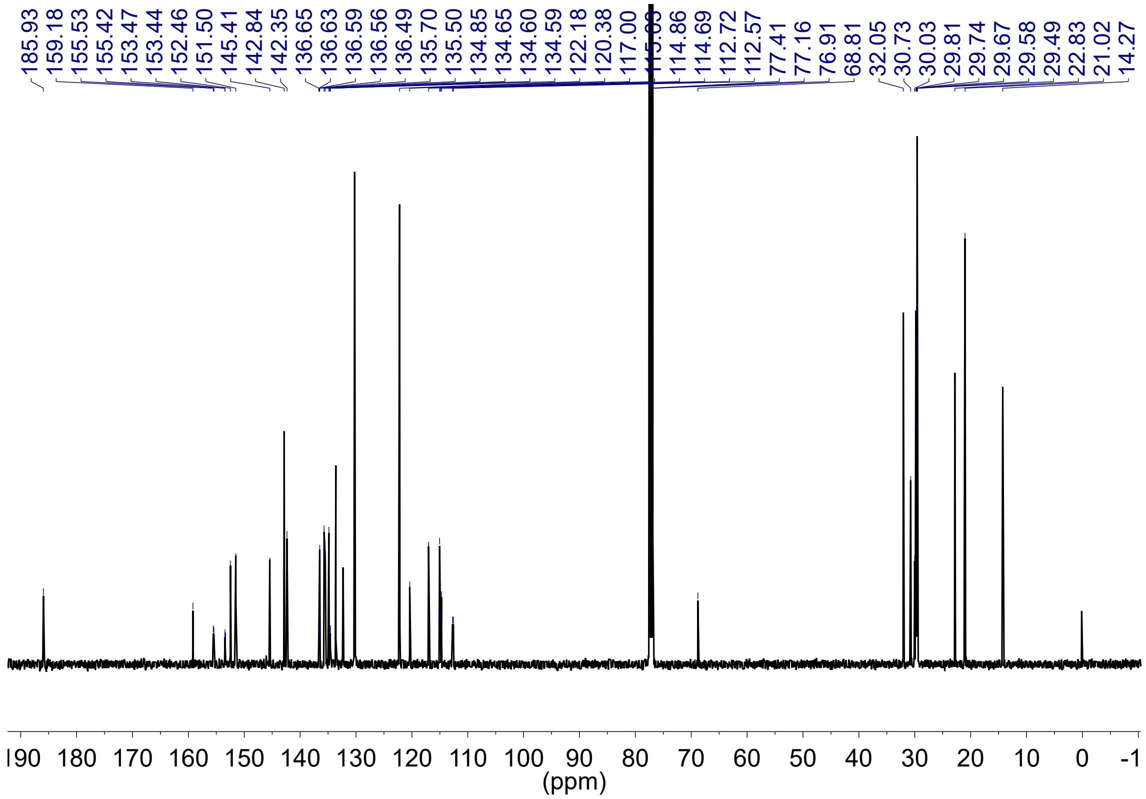

Figure S24 ^{13}C NMR spectrum of L0 in $CDCl_3$ solution.

References

1. D. L. Ma, Q. Q. Zhang and C. Z. Li, *Angew. Chem. Int. Ed.*, 2023, **62**, e202214931.
2. D. Neher, J. Kniepert, A. Elimelech and L. J. Koster, *Sci. Rep.*, 2016, **6**, 24861.
3. R. Zeng, M. Zhang, X. Wang, L. Zhu, B. Hao, W. Zhong, G. Zhou, J. Deng, S. Tan, J. Zhuang, F. Han, A. Zhang, Z. Zhou, X. Xue, S. Xu, J. Xu, Y. Liu, H. Lu, X. Wu, C. Wang, Z. Fink, T. P. Russell, H. Jing, Y. Zhang, Z. Bo and F. Liu, *Nat. Energy*, 2024, **9**, 1117–1128.
4. X. Zheng, W. Liu, N. Wei, A. Zhang, G. Ran, H. Shan, H. Huo, Y. Liu, H. Lu, X. Xu, Z. Tang, W. Zhang and Z. Bo, *Aggre.*, 2023, **5**, e469.
5. D. Li, H. Zhang, X. Cui, Y. N. Chen, N. Wei, G. Ran, H. Lu, S. Chen, W. Zhang, C. Li, Y. Liu, Y. Liu and Z. Bo, *Adv. Mater.*, 2024, **36**, e2310362.
6. X. Wang, R. Zeng, H. Lu, G. Ran, A. Zhang, Y. N. Chen, Y. Liu, F. Liu, W. Zhang, Z. Tang and Z. Bo, *Chin. J. Chem.*, 2023, **41**, 665-671.
7. X. Wang, H. Lu, Y. Liu, A. Zhang, N. Yu, H. Wang, S. Li, Y. Zhou, X. Xu, Z. Tang and Z. Bo, *Adv. Energy Mater.*, 2021, **11**, 2102591.
8. Z. Han, C. Zhang, T. He, J. Gao, Y. Hou, X. Gu, J. Lv, N. Yu, J. Qiao, S. Wang, C. Li, J. Zhang, Z. Wei, Q. Peng, Z. Tang, X. Hao, G. Long, Y. Cai, X. Zhang and H. Huang, *Angew. Chem. Int. Ed.*, 2024, **63**, e202318143.
9. X. Gu, R. Zeng, T. He, G. Zhou, C. Li, N. Yu, F. Han, Y. Hou, J. Lv, M. Zhang, J. Zhang, Z. Wei, Z. Tang, H. Zhu, Y. Cai, G. Long, F. Liu, X. Zhang and H. Huang, *Adv. Mater.*, 2024, **36**, e2401370.
10. Z. Xing, X. Wu, T. Chen, S. Ye, S. Wang, Y. Pan, S. Li, M. Shi and H. Chen, *J. Mater. Chem. A*, 2024, **12**, 11286-11294.
11. O. J. Sandberg and A. Armin, *PRX Energy*, 2024, **3**, 023008.
